# Clinical outcomes and remission trajectories in obese and non-obese patients with severe asthma treated with biologics: a retrospective longitudinal cohort study from the Severe Asthma Network Italy (SANI) registry

**DOI:** 10.1016/j.lanepe.2026.101695

**Published:** 2026-05-07

**Authors:** Danilo Di Bona, Giuseppe Di Gioia, Enrico Heffler, Roberta Parente, Gaetano Serviddio, Francesco Blasi, Pierluigi Paggiaro, Giorgio Walter Canonica, SANI network, Diego Bagnasco, Luisa Brussino, Cecilia Calabrese, Gianna Camiciottoli, Marco Caminati, Giovanna Elisiana Carpagnano, Cristiano Caruso, Angelo Guido Corsico, Maria Teresa Costantino, Claudia Crimi, Alice D'Adda, Simona D'Alò, Maria D'Amato, Leda D'Amico, Corrado D'Andria, Stefano Del Giacco, Fabiano Di Marco, Nicola Cosimo Facciolongo, Alessandro Farsi, Manuela Latorre, Manlio Milanese, Michele Mondoni, Eustachio Nettis, Vincenzo Patella, Girolamo Pelaia, Laura Pini, Luisa Ricciardi, Fabio Luigi Massimo Ricciardolo, Luca Richeldi, Erminia Ridolo, Pierachille Santus, Nicola Scichilone, Giulia Scioscia, Antonio Spanevello, Paolo Tarsia, Massimo Triggiani, Gilda Varricchi, Mona-Rita Yacoub

**Affiliations:** a Department of Medical and Surgical Sciences, University of Foggia, Italy; b Personalized Medicine, Asthma and Allergy, IRCCS Humanitas Research Hospital, Rozzano, Milan, Italy; c Department of Biomedical Sciences, Humanitas University, Pieve Emanuele, Milan, Italy; d Division of Allergy and Clinical Immunology, University of Salerno, Salerno, Italy; e Department of Pathophysiology and Transplantation, Università degli Studi di Milano, Italy; f Respiratory and Cystic Fibrosis Unit, Fondazione IRCCS Ca’ Granda Ospedale Maggiore Policlinico Milano, Italy; g Department of Surgery, Medicine, Molecular Biology and Critical Care, University of Pisa, Pisa, Italy

**Keywords:** Severe asthma, Obesity, Biologic therapy, Clinical remission, Real-world evidence

## Abstract

**Background:**

Obesity is common in severe asthma and associated with poorer control, reduced lung function, and comorbidities. Registry data suggest that obesity may lessen biologic effectiveness, but findings are inconsistent. We assessed the impact of obesity on long-term outcomes.

**Methods:**

We retrospectively analysed 2098 adults with severe asthma enrolled in the Italian Severe Asthma Network registry between 2017 and 2024. Patients were classified as biologic-naïve, initiating treatment during observation, or on-treatment at registry entry. Obesity was defined as body mass index (BMI) ≥30 kg/m^2^. Outcomes included exacerbations, Asthma Control Test (ACT), Asthma Quality of Life Questionnaire (AQLQ), Forced Expiratory Volume in 1 second (FEV_1_), and remission, defined as ≥12 consecutive months meeting prespecified criteria (no exacerbations or oral corticosteroids, ACT ≥ 20, FEV_1_ ≥ 80% predicted). Longitudinal changes were assessed using zero-inflated negative binomial and linear mixed-effects models; remission by Kaplan–Meier and Cox models.

**Findings:**

Of 2098 patients (1118 biologic-naïve; 980 on biologics at inclusion), 405 (19.3%) were obese. At baseline, obese patients showed poorer control, lower AQLQ, more comorbidities, but fewer nasal polyps and lower type-2 inflammation. During follow-up, exacerbations, ACT, AQLQ, and FEV_1_ improved similarly in obese and non-obese, without significant differences across naïve or on-treatment cohorts. At 24 months, cumulative remission probability was 22–25%. Obesity did not influence remission likelihood (HR for BMI ≥ 30: 0.88 [95% CI 0.57–1.37] in naïve; 0.91 [0.44–1.89] in on-treatment).

**Interpretation:**

Biologics remain efficacious in obese patients with severe asthma. Treatment achieved comparable improvement trajectories across all outcomes regardless of BMI, highlighting the importance of long-term follow-up.

**Funding:**

CN00000041_CN3_Spoke_#4_PNRR.

**Research in context**

**Evidence before this study**

Obesity is a common comorbidity in severe asthma, associated with worse disease control, reduced lung function, and greater multimorbidity. Several observational and registry studies have explored the impact of obesity on response to biologics, with some suggesting a lower likelihood of remission (as an emerging therapeutic goal), although findings remain inconsistent. We considered all available studies evaluating the association between obesity and clinical response or remission in adults with severe asthma treated with biologic therapies. We searched MEDLINE and Web of Science from 1 January 2000 to 1 January 2024, without language restrictions, using combinations of the following terms in Title/Abstract: “severe asthma”, “obesity”, “body mass index”, “biologics”, “biological therapy”, “anti-IgE”, “anti-IL5”, “anti-IL5R”, “anti-IL4/13”, “anti-TSLP”, “remission”, and “registry”. Reference lists of relevant articles and reviews were also screened. The search was updated during manuscript preparation to identify any additional relevant studies published up to 31 July 2025. The search yielded 382 publications, of which 214 remained after duplicate removal. We excluded editorials, background articles, guidelines, narrative reviews, case reports, mechanistic or pharmaco-economic studies, and studies addressing drugs or outcomes not relevant to our research question. Among the remaining 30 publications, we retained original observational studies and the most recent meta-analysis evaluating obesity as a predictor of response to biologic therapies in severe asthma. For the purpose of contextualising our findings, we identified 11 key retrospective real-world studies, including three large registry-based analyses, as well as one recent meta-analysis, addressing the potential negative impact of obesity on response to biologics. Several studies reported a reduced likelihood of achieving remission in obese patients, with a pooled odds ratio of 0.41 (95% CI 0.31–0.54) reported in the available meta-analysis, whereas other studies observed comparable improvements irrespective of body mass index. Overall, the available evidence consisted of studies with heterogeneous populations with variable follow-up durations, and the overall risk of bias was generally moderate to high. Definitions of remission varied across studies, and the 12-month observation period—generally regarded as the expert-consensus standard—was not uniformly applied, limiting comparability across studies. Moreover, longitudinal trajectories of exacerbations, asthma control, lung function, and quality of life under biologics have been only partly described. Consequently, the long-term impact of obesity on the course of severe asthma during biologic treatment remains incompletely understood.

**Added value of this study**

Our study provides, to our knowledge, the most comprehensive longitudinal assessment of the role of obesity in severe asthma under biologic therapy. We analysed 2098 patients enrolled in the Italian Severe Asthma Network (SANI), of whom 405 (19.3%) were obese. Several methodological features distinguish our study from previous work. First, outcomes were evaluated longitudinally at multiple prespecified time points rather than at a single snapshot, allowing us to capture trajectories over time. Second, remission was defined with a rigorous criterion requiring ≥12 consecutive months fulfilling all conditions (exacerbations, oral corticosteroid use, asthma control, stable FEV_1_) thereby excluding transient improvements and ensuring temporal consistency. Third, we combined two complementary cohorts: biologic-naïve patients, who provide insight into early treatment trajectories, and on-treatment patients, who allow assessment of long-term patterns after many years of biologic use. Finally, patients were observed for an extended period, with follow-up up to 48 months in both cohorts, offering one of the longest real-world evaluations of remission under biologics to date.

Our results show that, despite a clear baseline disadvantage—poorer asthma control, lower quality of life, and more comorbidities—obese patients experienced benefits from biologic therapy comparable to those observed in non-obese patients. Clinical outcomes, including exacerbations, asthma control, quality of life, and lung function, improved consistently across BMI categories, and the probability of achieving remission increased progressively during follow-up, with no evidence that obesity compromised the long-term effectiveness of biologics.

**Implications of all the available evidence**

Taken together with previous studies, our findings challenge the notion that obesity is a determinant of poor response to biologics in severe asthma. Instead, our longitudinal real-world evidence shows that obese patients can achieve meaningful improvements and remission following trajectories comparable to their non-obese counterparts, provided that follow-up is sufficiently long and outcomes are measured with rigorous definitions. For clinicians, this reinforces the importance of not excluding or deprioritising obese patients for biologic therapy, and of maintaining treatment over time even in the presence of complex multimorbidity. For researchers, our results highlight the need for longitudinal designs with repeated assessments rather than single-point evaluations, as compressed or heterogeneous observation windows may underestimate treatment success. Finally, for guideline developers and policymakers, these data support the use of biologics across BMI categories and underline the importance of registries capable of capturing extended follow-up.

## Introduction

Severe asthma is a heterogeneous and often debilitating condition that affects approximately 5–10% of individuals with asthma.^1^^,^^2^ It is associated with substantial morbidity, frequent exacerbations, poor quality of life, and high healthcare costs. The advent of biologic therapies targeting type 2 (T2) inflammation—such as anti-IgE, anti-IL5, anti-IL5R, anti-IL4/IL13, and anti-TSLP monoclonal antibodies—has significantly improved disease control and reduced reliance on systemic corticosteroids in most patients.^3^ In this evolving therapeutic landscape, the concept of clinical remission has emerged as a desirable treatment goal.^4^

Despite these advancements, interindividual variability in treatment response remains considerable, and the identification of factors influencing remission is a growing research priority.^5^ Among these, obesity has garnered increasing attention.^6^ Obesity is highly prevalent in patients with severe asthma, with estimates suggesting that more than 50% of this population may be obese.^7^ Obesity is not only a risk factor for asthma development and poor disease control but also appears to define a distinct asthma phenotype characterized by reduced response to inhaled corticosteroids, lower lung function, and higher healthcare utilization.^7^^,^^8^ Importantly, several national and international guidelines, including the A2BCD guide, emphasize the management of comorbidities such as obesity as a central component of comprehensive asthma care.^9^

Some recent real-world studies and registry-based analyses have reported higher body mass index (BMI) or obesity as negative predictors of clinical remission under biologic treatment, but findings remain inconsistent.^10^, ^11^, ^12^, ^13^ While patients with T2-high biomarkers are generally more likely to benefit from biologics, obesity has been associated in several studies with a reduced likelihood of achieving remission, regardless of the specific biologic agent used.^1^^,^^12^ Several mechanisms may underlie this observation, including altered pharmacokinetics and impaired drug distribution,^14^ obesity-associated systemic inflammation,^15^ and the presence of overlapping comorbidities such as obstructive sleep apnoea,^16^ or gastroesophageal reflux.^17^

To date, few studies have specifically investigated the impact of BMI on treatment outcomes in severe asthma.^18^ Our analysis, based on a large national cohort of nearly 2100 patients receiving biologic therapy within the Italian Registry of Severe Asthma (Severe Asthma Network Italy—SANI), aims to explore the relationship between obesity and clinical outcomes, with particular focus on remission rates in obese individuals.

## Methods

This retrospective, longitudinal study includes patients aged ≥12 years with severe asthma enrolled in the SANI registry, a nationwide network of specialized asthma centres across Italy, from January 2017 to December 31, 2024. Eligibility required fulfilment of ERS/ATS and GINA definitions of severe asthma, compliance with national reimbursement criteria for biologic therapy (Appendix p 2), availability of registry data before, or on, biologic initiation date for one or more study domain. All patients were included at baseline; only those with follow-up were analysed longitudinally. Post–biologic outcomes were evaluated at multiple time-points with maximum follow-up varying by outcome (up to 48 months).

The registry includes both patients who were already receiving biologic therapy at enrolment— having initiated treatment before registry inclusion—and biologic-naïve patients, starting biologics at the time of inclusion or during follow-up. Outcomes for both cohorts were compared with data from the year preceding registry inclusion. For both cohorts, changes were assessed longitudinally; however, a before–after design (pre- and post-biologic initiation) was specifically applied to biologic-naïve patients, whereas in on-treatment patients changes were evaluated over time from registry entry, using the preceding year as reference.

Patients with ≤1 month of biologic exposure at enrolment were classified as biologic-naïve, and those with >1 month as on-treatment. Despite the early improvements reported with some biologics —particularly benralizumab—,^19^ the one-month cut-off was chosen because such brief exposure is unlikely to influence outcomes, while classifying them as on-treatment would have increased heterogeneity without adding informative long-term data. In total, 71 patients were classified as biologic-naïve despite having initiated treatment within one month before enrolment (mean exposure 21.1 days [SD 9.5], range 2–31 days).

Baseline demographic (age, sex, BMI, smoking history) and asthma-related clinical characteristics (disease onset and duration, biomarkers, treatment, comorbidities) were recorded. Sex was recorded at baseline as reported in the clinical registry, based on biological sex. Race and ethnicity were recorded at baseline according to predefined registry categories, based on participant self-identification where available. Obesity was defined as BMI ≥ 30 kg/m^2^.

### Outcomes

Evaluated outcomes included: exacerbations requiring systemic corticosteroids, emergency department visits, hospitalizations, unscheduled visits, Asthma Control Test (ACT), Asthma Quality of Life Questionnaire (AQLQ), percent predicted pre-bronchodilator Forced Expiratory Volume in 1 s (FEV_1_%), and clinical remission.

All clinical, functional, and biological variables—including patient-reported outcomes (ACT, AQLQ), lung function (FEV_1_), exacerbations and oral corticosteroid use, biological markers (blood eosinophil counts, total IgE, and fractional exhaled nitric oxide [FeNO]), and comorbidities—were measured and recorded according to internationally recognised clinical standards and definitions, as part of routine clinical practice, in line with GINA and ERS/ATS recommendations.^1^^,^^2^ Clinical remission was defined according to the SANI Expert Consensus, with a slight modification to the lung function criterion (addition of pre-bronchodilator FEV_1_ ≥ 80% predicted to stable lung function).^20^ Remission required ≥12 consecutive months meeting the following: (1) no need for oral corticosteroid (OCS) treatment, (2) absence of asthma exacerbations or attacks, (3) absence of relevant asthma symptoms (ACT ≥ 20), and (4) stable lung function with pre-bronchodilator FEV_1_ ≥ 80% predicted. Complete remission required all four criteria; partial remission required absence of oral corticosteroid use plus any two of the remaining three (Appendix p 3).^20^

A T2-high profile was defined by ≥2 of 3 biomarkers: FeNO ≥ 25 ppb, blood eosinophils ≥300 cells/μL, or total serum IgE ≥ 100 IU/mL (Appendix p 3).

### Statistical methods

No formal sample size calculation was performed; all available registry data were used. Group characteristics were compared using Pearson's chi-square test for categorical variables and binary data, and Student's t-test for normally distributed continuous variables.

The Wilcoxon rank-sum test was used for non-normally distributed continuous variables, and for follow-up time comparisons.

Zero-inflated negative binomial (ZINB) models assessed associations between BMI and counts of exacerbations, hospitalizations, unplanned visits, and emergency department admissions, accounting for overdispersion and excess zeros in the count data.

Mixed-effects models evaluated associations between BMI and ACT, AQLQ, and FEV1, incorporating both fixed and random effects to account for within-subject variability over time.

Kaplan–Meier curves estimated the probability of achieving clinical remission over time in obese vs. non-obese patients; groups were compared using the log-rank test. By definition remission could only be observed after ≥12 months of follow-up, meaning that the earliest time at which remission could be assigned was after 12 months of follow-up. Missing data within the first 12 months (patients not yet observed, loss to follow-up, or deceased) were explored to determine whether they reflected differential non-response between BMI categories (<30 vs. ≥30), thereby minimising the risk of attrition bias between groups.

Cox proportional hazards models evaluated the association between BMI and partial or complete remission in patients without remission at baseline. The proportional hazards assumption was verified using the Schoenfeld Residuals Test. Given the substantial proportion of missing data across treatment groups and BMI categories, sensitivity analyses using multiple imputation by chained equations (MICE) were undertaken to evaluate the robustness of the primary findings in a larger analytic sample with increased statistical power (Appendix p 34).

Sensitivity analyses modelled BMI as a continuous variable in ZINB and mixed-effects models. To aid interpretation, post-estimation margins were used to derive predicted values at representative BMI levels, which were plotted graphically. BMI was also modelled as a continuous variable in Cox regression.

All models were adjusted for age, sex, presence of chronic rhinosinusitis with nasal polyps (CRSwNP), blood eosinophil count, FEV_1_ (% predicted), ACT score, and days on biologic therapy at baseline, selected based on literature evidence of potential influence on the outcome.

Stata version 15.0 SE (StataCorp, College Station, TX, USA) was used to perform the analyses.

### Ethics approval

The registry has been described elsewhere and has ethical approval for collecting, storing, analysing, and reporting anonymised data with each patient’s written consent.^21^ The protocol is registered at ClinicalTrials.gov (ID NCT06625216; retrospectively registered on October 3, 2024).

### Role of the funding source

This work was supported by institutional funds from the European Union (EU) within the Italian Ministry of University and Research (MUR) National Recovery and Resilience Plan (PNRR; project CN00000041, CN3 Spoke #4 NRR MUR—M4C2 ‘Metabolic and cardiovascular diseases’), which were used for the acquisition of software licenses and computing equipment necessary for data management and analysis. The funding source had no role in the study design, data collection, data analysis, data interpretation, or writing of the report.

## Results

As of December 31, 2024, 3193 patients had been enrolled in the SANI registry; 2098 met all inclusion criteria: 1118 were biologic-naïve at inclusion (initiated biologic therapy at or after enrolment), and 980 were already receiving biologics (on-treatment) (Appendix p 4; Table 1). The main reasons for exclusion were absence of biologic initiation during the study period (n = 799, controlled on high-dose inhaled therapy) and lack of biological exposure during follow-up (n = 94).

**Table 1:** Baseline characteristics of the study population by BMI status.

	Biologic-naïve at inclusion				On biologics at inclusion			
	Total	BMI < 30	BMI ≥ 30	p	Total	BMI < 30	BMI ≥ 30	p
	N = 1118	N = 893	N = 225		N = 980	N = 800	N = 180	
Age	56.00 (48.00, 64.00)	55.00 (47.00, 64.00)	58.00 (50.00, 66.00)	**0.021**	58.00 (49.00, 65.00)	57.00 (49.00, 64.00)	61.00 (53.00, 67.00)	**0.002**
Sex (Female)	684 (61.18%)	531 (59.46%)	153 (68.00%)	**0.019**	612 (62.45%)	485 (60.62%)	127 (70.56%)	**0.013**
BMI	26.22 (5.06)	24.28 (3.10)	33.94 (3.83)	**<0.001**	26.13 (4.90)	24.37 (3.03)	33.94 (3.89)	**<0.001**
Current smokers	49 (4.42%)	37 (4.18%)	12 (5.36%)	0.068	32 (3.30%)	28 (3.53%)	4 (2.29%)	0.39
Former smokers	324 (29.22%)	246 (27.80%)	78 (34.82%)		239 (24.66%)	201 (25.31%)	38 (21.71%)	
Never smokers	736 (66.37%)	602 (68.02%)	134 (59.82%)		698 (72.03%)	565 (71.16%)	133 (76.00%)	
Onset asthma (age)	35.00 (20.00, 47.00)	34.00 (20.00, 46.00)	38.00 (20.00, 50.00)	0.11	33.00 (20.00, 45.00)	34.00 (20.00, 45.00)	30.00 (16.50, 45)	0.39
Diagnosis asthma (age)								
<18 (n, %)	172 (20.98)	141 (21.66)	31 (18.34)	0.261	186 (21.16)	146 (20.19)	40 (25.64)	0.113
18–39 (n, %)	300 (36.59)	243 (37.33)	57 (33.73)		354 (40.27)	302 (41.77)	52 (33.33)	
≥40 (n, %)	348 (42.44)	267 (41.01)	81 (47.93)		339 (38.57)	275 (38.04)	64 (41.03)	
Clinical status								
ACQ >1.5 or ACT <20 or uncontrolled according to GINA	690 (74.84%)	549 (74.09%)	141 (77.90%)	0.29	553 (56.43%)	446 (55.75%)	107 (59.44%)	0.37
ACT	15.06 ( × /1.45)	15.36 ( × /1.44)	13.94 ( × /1.48)	**<0.001**	19.05 ( × /1.35)	19.25 ( × /1.34)	18.14 ( × /1.38)	**0.025**
AQLQ	3.99 ( × /1.40)	4.04 ( × /1.40)	3.79 ( × /1.37)	**0.032**	4.90 ( × /1.37)	4.95 ( × /1.37)	4.68 ( × /1.37)	0.073
FEV1 post-bronchodilator <80% of predicted	284 (30.80%)	224 (30.23%)	60 (33.15%)	0.45	301 (30.71%)	250 (31.25%)	51 (28.33%)	0.44
FEV1% pre-bronchodilator	74.00 (58.00, 89.00)	75.00 (58.00, 90.00)	71.00 (56.00, 83.00)	0.11	77.00 (65.00, 93.00)	77.00 (65.00, 93.00)	76.00 (66.00, 96.00)	0.83
FEV1% post-bronchodilator	80.00 (66.00, 95.00)	80.00 (66.20, 96.00)	79.50 (60.00, 92.10)	0.21	82.00 (69.30, 95.00)	82.00 (70.00, 95.00)	82.75 (66.00, 95.00)	0.72
Patients with ≥2 exacerbations treated with systemic CS > 3 consecutive days in the year before biologic	516 (55.97%)	423 (57.09%)	93 (51.38%)	0.17	471 (48.06%)	401 (50.13%)	70 (38.89%)	**0.006**
Exacerbations requiring CS				0.78				0.25
0	275 (24.60%)	217 (24.30%)	58 (25.78%)		485 (49.49%)	405 (50.63%)	80 (44.44%)	
1	125 (11.18%)	104 (11.65%)	21 (9.33%)		131 (13.37%)	103 (12.88%)	28 (15.56%)	
2–5	517 (46.24%)	411 (46.02%)	106 (47.11%)		250 (25.51%)	205 (25.62%)	45 (25.00%)	
>5	201 (17.98%)	161 (18.03%)	40 (17.78%)		114 (11.63%)	87 (10.88%)	27 (15.00%)	
At least one hospitalization, ICU admission, or mechanical ventilation for asthma in the past year	64 (6.94%)	54 (7.29%)	10 (5.52%)	0.40	54 (5.51%)	46 (5.75%)	8 (4.44%)	0.49
Asthma related hospitalizations				0.57				0.19
0	914 (81.75%)	734 (82.19%)	180 (80.00%)		845 (86.22%)	694 (86.75%)	151 (83.89%)	
1	121 (10.82%)	93 (10.41%)	28 (12.44%)		63 (6.43%)	47 (5.88%)	16 (8.89%)	
2–5	34 (3.04%)	29 (3.25%)	5 (2.22%)		25 (2.55%)	23 (2.88%)	2 (1.11%)	
>5	49 (4.38%)	37 (4.14%)	12 (5.33%)		47 (4.80%)	36 (4.50%)	11 (6.11%)	
Asthma related ED visits				0.74				0.14
0	856 (76.57%)	690 (77.27%)	166 (73.78%)		808 (82.45%)	666 (83.25%)	142 (78.89%)	
1	113 (10.11%)	87 (9.74%)	26 (11.56%)		65 (6.63%)	50 (6.25%)	15 (8.33%)	
2–5	92 (8.23%)	72 (8.06%)	20 (8.89%)		40 (4.08%)	35 (4.38%)	5 (2.78%)	
>5	57 (5.10%)	44 (4.93%)	13 (5.78%)		67 (6.84%)	49 (6.13%)	18 (10.00%)	
No scheduled visits				0.80				**0.004**
0	555 (49.64%)	438 (49.05%)	117 (52.00%)		681 (69.49%)	568 (71.00%)	113 (62.78%)	
1	60 (5.37%)	47 (5.26%)	13 (5.78%)		44 (4.49%)	32 (4.00%)	12 (6.67%)	
2–5	252 (22.54%)	206 (23.07%)	46 (20.44%)		114 (11.63%)	98 (12.25%)	16 (8.89%)	
>5	251 (22.45%)	202 (22.62%)	49 (21.78%)		141 (14.39%)	102 (12.75%)	39 (21.67%)	
On treatment with:								
High doses of inhaled corticosteroids plus LABA or another controller	851 (92.30%)	685 (92.44%)	166 (91.71%)	0.74	935 (95.41%)	763 (95.38%)	172 (95.56%)	0.92
OCS for at least six months in the past year	296 (32.10%)	238 (32.12%)	58 (32.04%)	0.98	217 (22.14%)	181 (22.63%)	36 (20.00%)	0.44
Daily prednisone-equivalent dosage in the year before baseline	6.70 (5.00, 25.00)	6.70 (5.00, 25.00)	16.54 (5.00, 25.00)	0.68	5.00 (5.00, 25.00)	5.00 (5.00, 25.00)	16.00 (5.00, 25.00)	0.59
Biomarkers								
FeNO	34.03 ( × /2.67)	37.32 ( × /2.59)	23.53 ( × /2.77)	**<0.001**	28.20 ( × /2.54)	28.48 ( × /2.56)	26.73 ( × /2.48)	0.60
Eosinophils (n)	0.41 (0.20, 0.70)	0.43 (0.20, 0.71)	0.36 (0.14, 0.64)	**0.033**	0.20 (0.08, 0.50)	0.20 (0.08, 0.51)	0.23 (0.07, 0.49)	0.96
Neutrophils (n)	4.03 (3.12, 5.20)	3.90 (3.06, 5.14)	4.53 (3.53, 5.47)	**0.002**	3.90 (3.11, 5.12)	3.90 (3.11, 5.12)	4.07 (3.16, 5.14)	0.80
Total IgE	186.00 (80.70, 423.00)	188.00 (86.00, 421.00)	181.00 (62.50, 424.00)	0.33	227.00 (88.60, 538.00)	228.00 (85.00, 527.00)	211.80 (108.00,687.00)	0.43
Th2 profile				**0.023**				0.35
Low	516 (46.15%)	397 (44.46%)	119 (52.89%)		503 (51.33%)	405 (50.63%)	98 (54.44%)	
High	602 (53.85%)	496 (55.54%)	106 (47.11%)		477 (48.67%)	395 (49.38%)	82 (45.56%)	
Comorbidities								
Allergy	644 (57.60%)	512 (57.33%)	132 (58.67%)	0.72	668 (68.16%)	545 (68.13%)	123 (68.33%)	0.96
Rhinitis				0.53				0.49
Never	412 (37.18%)	327 (36.91%)	85 (38.29%)		340 (35.20%)	273 (34.47%)	67 (38.51%)	
Yes, ongoing	578 (52.17%)	460 (51.92%)	118 (53.15%)		464 (48.03%)	382 (48.23%)	82 (47.13%)	
Yes, previous	118 (10.65%)	99 (11.17%)	19 (8.56%)		162 (16.77%)	137 (17.30%)	25 (14.37%)	
CRSsNP	309 (28.19%)	247 (28.23%)	62 (28.05%)	0.96	261 (27.13%)	226 (28.61%)	35 (20.35%)	**0.027**
CRSwNP	526 (47.13%)	449 (50.39%)	77 (34.22%)	**<0.001**	444 (45.77%)	377 (47.42%)	67 (38.29%)	**0.028**
OSAS	67 (6.37%)	30 (3.57%)	37 (17.45%)	**<0.001**	41 (4.39%)	21 (2.74%)	20 (11.90%)	**<0.001**
GI reflux	414 (37.67%)	315 (35.96%)	99 (44.39%)	**0.020**	369 (38.20%)	293 (36.99%)	76 (43.68%)	0.10
Cardiovascular disease	320 (30.19%)	211 (25.03%)	109 (50.23%)	**<0.001**	279 (30.53%)	196 (26.17%)	83 (50.30%)	**<0.001**
Type 2 diabetes	49 (4.62%)	29 (3.43%)	20 (9.30%)	**<0.001**	49 (5.34%)	27 (3.60%)	22 (13.02%)	**<0.001**
Osteoporosis	131 (13.60%)	103 (13.41%)	28 (14.36%)	0.73	135 (15.73%)	111 (15.68%)	24 (16.00%)	0.92
Mood disorders	107 (10.09%)	80 (9.47%)	27 (12.56%)	0.18	90 (9.83%)	72 (9.64%)	18 (10.65%)	0.69

Data are presented as medians ± interquartile range (IQR), harmonic means ( × /multiplicative SD) or numbers (%). P values indicating statistical significance are presented in bold.

BMI, body mass index; ACT, Asthma Control Test; ACQ, Asthma Control Questionnaire; GINA, Global Initiative for Asthma; AQLQ, Asthma Quality of Life Questionnaire; FEV_1_, forced expiratory volume in 1 second; CS, corticosteroid; ICU, intensive care unit; ED, emergency department; LABA, long-acting betaagonist; OCS, oral corticosteroids; FeNO, Fractional Exhaled Nitric Oxide; IgE, immunoglobulin E; CRSsNP, chronic rhinosinusitis without nasal polyps; CRSwNP, chronic rhinosinusitis with nasal polyps; OSAS, obstructive sleep apnoea syndrome; GI, gastrointestinal.

On-treatment patients represented a heterogeneous group, as they had been exposed to biologics for varying durations (mean 935.6 [SD 1111.6] days; Appendix p 5). Consequently, baseline data (referring to the year preceding inclusion) were influenced by ongoing biologic therapy to a variable extent depending on the duration of treatment prior to enrolment.

At baseline, 641 (31%) patients were receiving anti-IgE, 1151 (55%) anti-IL5/R, 248 (12%) anti-IL4/13, and 49 (2%) anti-TSLP, with no significant differences observed across BMI categories (Appendix p 6).

Baseline characteristics are reported in Table 1 (data completeness in Appendix p 7–10). Overall, race and ethnicity data were available for 1718 of the 2098 included patients (81.9%); of these, 1661 (96.7%) were classified as Caucasian according to registry categories.

Patients were stratified by BMI (<30 vs. ≥30) within the biologic-naïve and on-treatment groups.

Biologic-naïve patients were slightly younger compared to those already on biologics (median age 56 [IQR 48; 64] vs. 58 [49; 65] years), with higher age among patients with BMI ≥ 30 in both groups. Female sex was more frequent in obese patients (68.0% vs. 59.5% in naïve; 70.6% vs. 60.6% in on-treatment).

Asthma control and quality of life were worse in naïve patients compared with those already on biologics, and lower in obese patients in both groups (ACT: 13.9 vs. 15.4 in naïve [p < 0.001]; 18.1 vs. 19.3 in on-treatment [p = 0.025]; AQLQ: 3.8 vs. 4.0 in naïve [p = 0.032]; 4.7 vs. 5.0 in on-treatment [p = 0.073]).

Pre- and post-bronchodilator FEV_1_ did not differ by BMI.

The number of exacerbations per patient was comparable across BMI categories in both cohorts, but the proportion of patients with ≥2 exacerbations in the preceding year was higher among biologic-naïve than on-treatment patients (56.0% vs. 48.1%). Interpretation of this difference is limited by the absence of baseline exacerbation data and the heterogeneity in treatment duration in the on-treatment group. Among biologic-naïve patients, obesity was associated with a non-significant trend toward fewer individuals experiencing ≥2 events (51.4% vs. 57.1%, p = 0.17), whereas in the on-treatment cohort the difference was statistically significant (38.9% vs. 50.1%, p = 0.006).

Daily prednisone-equivalent dose was slightly lower in on-treatment patients but did not differ by BMI (naïve, p = 0.68; on-treatment, p = 0.59).

Biologic-naïve obese patients had lower FeNO levels than non-obese patients (23.5 vs. 37.3 ppb, p = 0.001). Blood eosinophil counts were also significantly lower in obese patients (0.36 vs. 0.43 × 10^9^/L; p = 0.03). The prevalence of a T2-high profile was greater in non-obese biologic-naïve patients (p = 0.023), while no differences by BMI were observed in the on-treatment group. Most patients had a positive allergy test to inhalants, with no BMI-related differences.

Comorbidities were more common among obese patients, particularly obstructive sleep apnoea (OSAS), gastro-oesophageal reflux disease (GERD), cardiovascular disease, and type 2 diabetes (all p < 0.001, except GERD in the on-treatment group). Conversely, chronic rhinosinusitis with nasal polyps (CRwNP) was less frequent in obese patients.

**Fig. 1:**
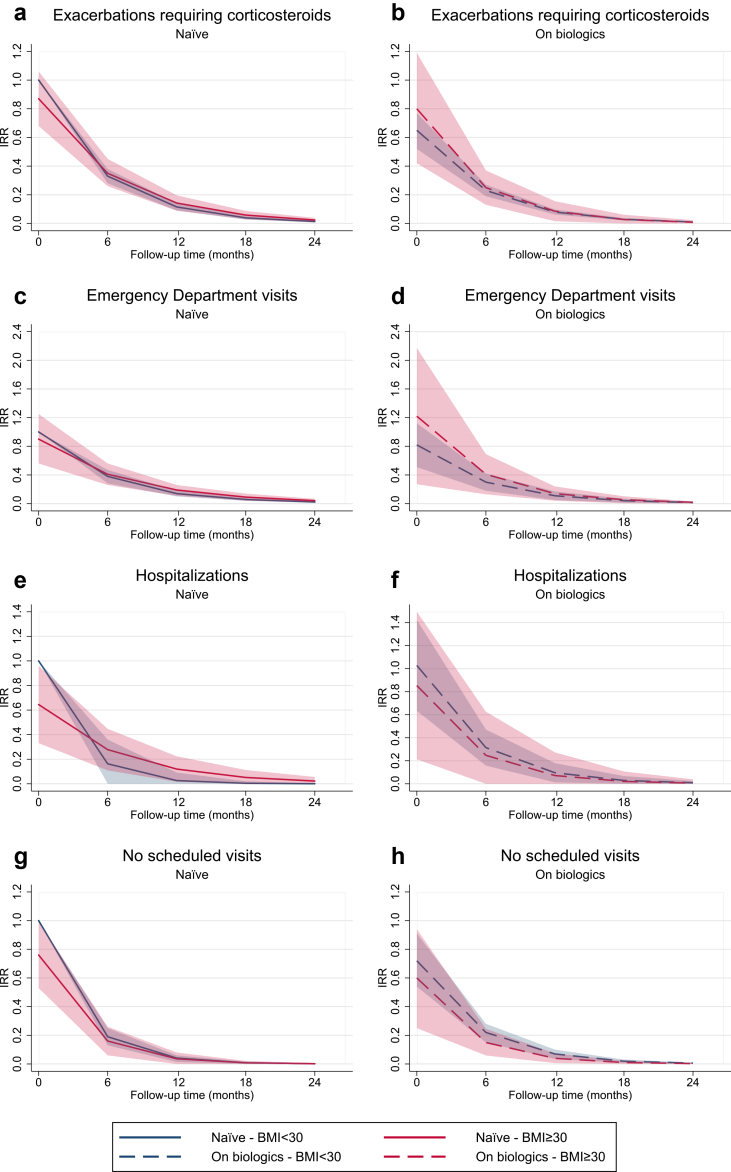
**Predicted rate of asthma exacerbationsand exacerbation-related outcomes (emergency department visits, hospitalitations, unscheduled visits)over time by treatment group and BMI category.** Longitudinal estimates of the outcomes are shown separately for biologic-naïve patients (a, c, e, g) and those already receiving biologics (b, d, f, h), stratified by BMI category (<30 vs. ≥30 kg/m^2^). Models were fitted using zero-inflated negative binomial (ZINB) regression and adjusted for baseline age, sex, presence of chronic rhinosinusitis with nasal polyps (CRSwNP), blood eosinophil count, FEV_1_ (% predicted), ACT score, and number of days on biologic therapy at baseline. The reference group consists of naïve patients with BMI <30 at baseline. Shaded areas represent 95% confidence intervals. IRR, incidence rate ratio.

Over 24 months, zero-inflated negative binomial models adjusted for relevant covariates (age, sex, ACT, FEV_1_, blood eosinophils, CRwNP, and treatment duration for on-treatment patients) showed no clinically meaningful differences between obese and non-obese patients in exacerbations requiring corticosteroids, asthma-related emergency visits, hospitalisations, or unscheduled visits (Fig. 1; Appendix p 11–14). These findings indicate that outcome rates declined substantially over time either in biologic-naïve or on-treatment cohorts, irrespective of BMI.

**Fig. 2:**
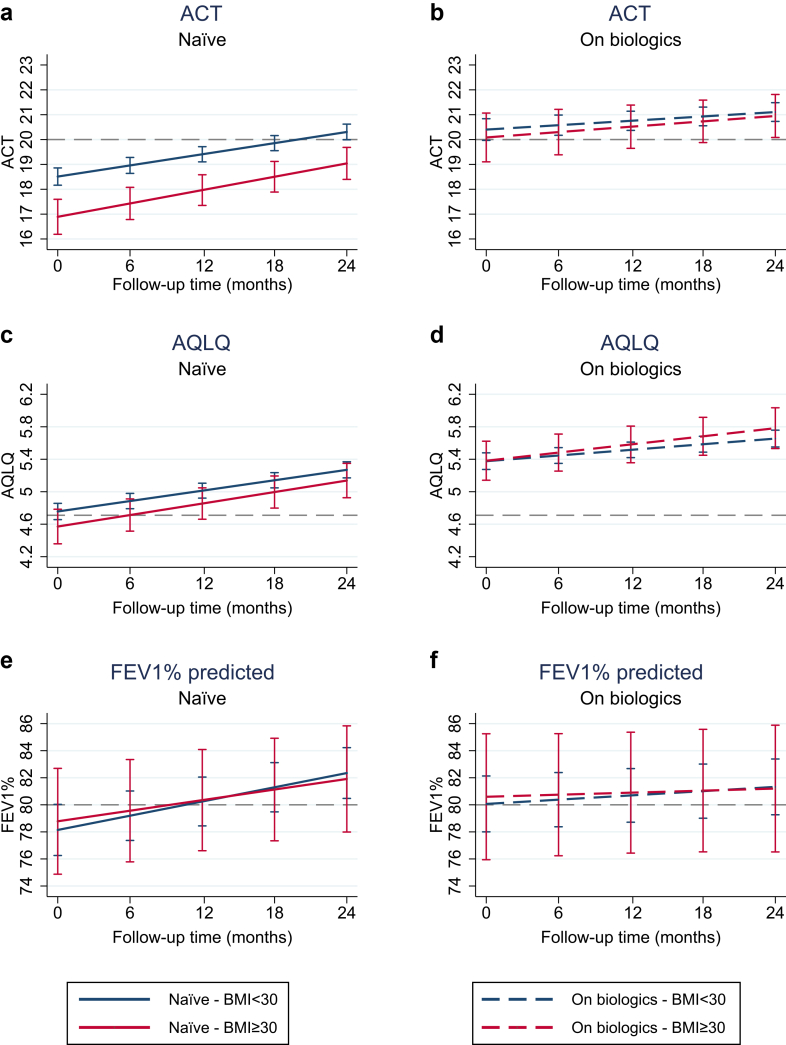
**Predicted changes in ACT score, AQLQ, and FEV_1_% over time by treatment group and BMI category.** Linear mixed-effects models were used to estimate changes in Asthma Control Test (ACT) scores, Asthma Quality of Life Questionnaire (AQLQ) scores, and percent predicted forced expiratory volume in 1 second (FEV_1_) over 24 months of follow-up. Analyses were stratified by BMI category (<30 vs. ≥30 kg/m^2^) and conducted separately for biologic-naïve patients (a, c, e) and those receiving biologics (b, d, f). Models were adjusted for baseline age, sex, presence of chronic rhinosinusitis with nasal polyps (CRSwNP), baseline blood eosinophil count, baseline ACT score, number of exacerbations in the previous year, and number of days on biologic therapy at baseline. Error bars represent 95% confidence intervals.

Linear mixed-effects models estimating longitudinal changes in ACT, AQLQ, and FEV_1_% predicted confirmed similar longitudinal trajectories across BMI categories (Fig. 2) in both groups (naïve vs. on-treatment). In biologic-naïve patients, ACT scores were consistently lower in obese individuals, but the difference did not reach the threshold of clinical relevance (≥3 points).^22^ AQLQ and FEV_1_% showed overlapping confidence intervals and no consistent differential pattern by BMI. In on-treatment patients, trajectories were flatter and remained within a narrow range regardless of BMI, with higher baseline values reflecting previous treatment benefits (corresponding model estimates and confidence intervals in Appendix, p15–16).

Sensitivity analyses for ZINB and mixed-effects models with BMI as a continuous variable yielded consistent results, with no differential trajectories observed across BMI strata for exacerbations, healthcare utilisation, ACT, AQLQ, or FEV_1_ (corresponding estimates in Appendix, p 17–25).

Kaplan–Meier analyses, restricted to patients not in remission at baseline estimated the time to partial or complete remission. At enrolment, partial or complete remission was observed in 20.4% of biologic-naïve and 46.7% of on-treatment patients, with no BMI-related differences (Appendix p 26). These patients were, therefore, not considered at risk in survival analysis.

**Fig. 3:**
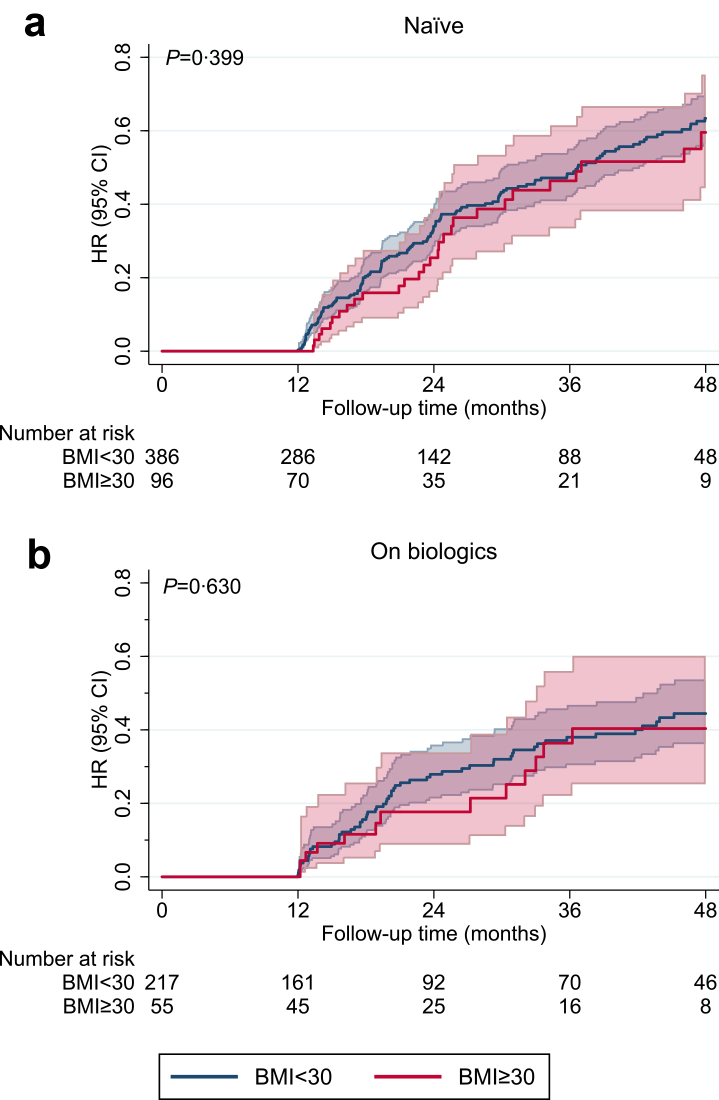
**Partial or complete clinical remission stratified by BMI and treatment status.** Kaplan–Meier curves show the estimated probability of achieving partial or complete clinical remission over time among patients stratified by BMI category (<30 vs. ≥30 kg/m^2^) and biologic treatment status (naïve [a] vs. on biologics [b]). The analysis included only patients who had not achieved remission at baseline. Shaded areas indicate 95% confidence intervals. The p value corresponds to the log-rank test for between-group comparison. HR, hazard ratio.

Kaplan–Meier curves showed no significant difference in time to partial or complete remission between obese and non-obese patients, in either biologic-naïve or on-treatment cohorts (Fig. 3). At 24 months, the cumulative probability of remission was 22–25% across BMI strata, with overlapping 95% CIs. The early plateau observed in the first 12 months reflected the requirement for 12 consecutive months fulfilling remission criteria and possible early dropouts. Dropout rates and adverse events did not differ by BMI (Appendix p 27–28), excluding differential attrition as a source of bias (i.e., the reduction in patients at risk between 0 and 12 months did not disproportionately affect the obese group). Follow-up time did not differ across BMI categories in either group (naïve 671.5 vs. 651 days, p = 0.151; on-treatment 761 vs. 718 days, p = 0.439; Appendix p 29).

Multivariable Cox regression models adjusted for age, sex, ACT, FEV_1_, blood eosinophils, CRwNP, and baseline exacerbations (plus treatment duration in already-treated patients) indicated that obesity did not influence the likelihood of remission (naïve: HR 0.88 [95% CI 0.57–1.37]; p = 0.586; on-treatment: HR 0.91 [0.44–1.89]; p = 0.806; Table 2). Similar results were obtained when modelling BMI as a continuous variable (naïve: HR 0.99 [95% CI 0.95–1.02]; p = 0.450; on-treatment: HR 0.95 [0.89–1.01]; p = 0.104; Table 2), and by sensitivity analysis accounting for centre-level variability, using a shared frailty term (Appendix p 29). Two further sensitivity analyses supported the robustness of the findings: (1) excluding the 71 patients who had initiated biologic therapy within one month before enrolment did not modify the estimates (Appendix p 29), and (2) starting the risk period at 12 months (i.e., when remission can first occur by definition) produced similar results, indicating that immortal time bias was not present (Appendix p 30).

**Table 2:** Cox models estimated Hazard Ratios of incident partial or complete remission, stratified by treatment group.

	Naive		On biologics	
	HR (95% CI)	p	HR (95% CI)	p
BMI ≥ 30	0.88 (0.57, 1.37)	0.586	0.91 (0.44, 1.89)	0.806
Age	1.00 (0.98, 1.01)	0.686	1.00 (0.97, 1.02)	0.794
Sex (Female)	0.98 (0.69, 1.39)	0.929	0.70 (0.41, 1.18)	0.184
CRwNP	0.94 (0.68, 1.31)	0.736	1.26 (0.68, 1.89)	0.653
BEC	1.02 (0.99, 1.05)	0.111	0.78 (0.41, 1.49)	0.453
FEV_1_% (pre-bronchodilator)	1.00 (1.00, 1.01)	**0.046**	1.00 (0.99, 1.01)	0.635
ACT	0.99 (0.96, 1.03)	0.719	1.04 (0.99, 1.10)	0.127
Exacerbations	0.99 (0.92, 1.05)	0.681	1.00 (0.85, 1.16)	1.000
Days on biologic therapy	-	-	1.00 (0.99, 1.00)	0.134

	Naive		On biologics	
	HR (95% CI)	p	HR (95% CI)	p
**BMI**	0.99 (0.95, 1.02)	0.450	0.95 (0.89, 1.01)	0.104

BMI was analysed both as a categorical variable (≥30 vs. <30 kg/m^2^) and as a continuous variable (results shown in the lower panel of the table). P values indicating statistical significance are presented in bold.

BMI, body mass index; CRwNP, chronic rhinosinusitis with nasal polyps; BEC, blood eosinophil count; FEV_1_%, forced expiratory volume in 1 second.

Another sensitivity analysis modelling BMI as a continuous variable using restricted cubic splines showed no evidence of non-linear associations between BMI and remission, with spline-derived estimates remaining close to unity (Appendix p 31), further supporting the absence of a meaningful BMI effect. Consistent findings were also obtained when patients were stratified by BMI categories (<25, 25–29, ≥30) (Appendix p 32), showing a non-significant trend toward better outcomes in patients with normal weight compared with those who were overweight or obese, particularly among patients already on biologic therapy. A separate sensitivity analysis stratifying patients by smoking status showed no significant differences in remission trajectories across smoking categories (Appendix p 33).

Finally, to assess the potential influence of missing data on our results, we performed multiple imputation as an additional sensitivity analysis (Appendix p 34–35). The imputed models yielded effect estimates that were consistent with the complete-case analyses, with no influence of obesity on the likelihood of remission, thereby confirming the robustness of the original findings on a larger analytic sample (Appendix p 35).

## Discussion

In this large real-world analysis of nearly 2100 patients from the Italian Severe Asthma Registry (SANI), obesity— although associated with a distinct clinical and inflammatory profile—did not significantly affect the long-term effectiveness of biologic therapy in severe asthma. Over time, both obese and non-obese patients showed consistent improvements in key outcomes, with comparable reductions in exacerbations (Fig. 1), better ACT and AQLQ scores, and improved FEV_1_ (Fig. 2). Although ACT scores remained slightly lower in obese naïve patients, the difference was clinically negligible, and trajectories were largely parallel.^22^ Remission accrued progressively in both groups, often only after two or more years of sustained treatment, highlighting the importance of extended follow-up to capture the full therapeutic benefit (Fig. 3). These findings proved robust across multiple sensitivity analyses (BMI modelled as a continuous variable, including with restricted cubic splines; stratification into three BMI categories; stratification by smoking status; and adjustment for centre-level clustering).

These results are noteworthy given the baseline disadvantage of obese patients, who presented with poorer asthma control, reduced quality of life, and more comorbidities, such as OSAS, GERD, cardiovascular disease, and type 2 diabetes. Such conditions complicate disease management, limiting the therapeutic window for certain drugs, and are usually linked to poorer outcomes.^17^^,^^23^^,^^24^ Similarly, baseline differences in T2 biomarkers did not translate into differential treatment responses. Obese patients, although belonging to a population with high T2 inflammation and eligible for biologic therapy, showed a blunted T2-high signature, lower prevalence of CRwNP and higher neutrophil counts—particularly evident in the biologic-naïve cohort, where biomarker levels were unaffected by prior treatment. Nevertheless, this attenuation of T2 inflammation was not sufficient to compromise the effectiveness of targeted biologics.^25^^,^^26^ It might, however, help explain the slightly lower proportion of obese patients experiencing ≥2 exacerbations, a trend significant only in the on-treatment cohort, since a blunted T2 profile could plausibly reduce susceptibility to acute exacerbations once adequate disease control is achieved.

Our findings contrast with some registry-based studies, such as the Danish Severe Asthma Register (DSAR) and the UK Severe Asthma Registry (UKSAR), which reported obesity as a negative predictor of remission at annual review.^10^^,^^11^ A subsequent UKSAR analysis refined the initial observation, showing that biologics were effective across all BMI groups, with attenuated responses in severely obese patients largely attributable to their worse baseline profile; however, that study assessed outcomes such as exacerbations and asthma control, not remission.^18^ A recent meta-analysis of observational studies suggested a significant negative effect of obesity on remission, although with substantial heterogeneity in outcomes and observation windows.^12^ By contrast, the International Severe Asthma Registry (ISAR) did not identify obesity as a predictor of treatment response.^13^

A key methodological distinction from other observational and registry-based analyses is our stricter and temporally consistent definition of remission, requiring all criteria (absence of OCS use, no exacerbations, controlled symptoms, and FEV_1_ ≥ 80% predicted) to be fulfilled at each assessment over 12 consecutive months. Other registries have also attempted to capture stability, but with less consistent timeframes. In UKSAR, remission is defined at the time of annual review, which may occur 9–24 months after the previous assessment. This variable schedule introduces heterogeneity: intervals shorter than 12 months (e.g., 9 months) fail to demonstrate sustained disease control over a full year, whereas longer intervals (e.g., 18–24 months) may overestimate stability by assuming continuous control throughout. In ISAR, exacerbations are reported as an annualized rate, based on events assessed within a variable follow-up window of 48–80 weeks after biologic initiation, while the other domains—ACT, FEV_1_, OCS use—are derived from the visit closest to 1 year after biologic initiation, provided this occurred between 24 and 80 weeks. Both approaches contrast with our stricter and temporally consistent definition of remission, which requires all domains to be fulfilled at each assessment over 12 consecutive months.^10^^,^^13^ Variability in definition of remission was also found in the studies included in the meta-analysis by Shackleford et al. which identified as many as 48 different definitions of clinical remission across studies.^12^ For these reasons, our estimates at 24 months are not directly comparable with those derived from other studies.^10^, ^11^, ^12^, ^13^ Using time-to-event methods, we observed cumulative rates of about 22–25% by 24 months, noting that remission in our analysis could not, by definition, be ascertained before 12 months (Fig. 3). Reported rates in other registries are numerically similar (18–20%) but are derived from annual reviews, performed at variable intervals, as previously noted.

Conversely, our analysis encompassed both partial and complete remission, which may further contribute to heterogeneity and may partly explain the higher estimates observed in our analysis compared with studies applying either definition alone.

The lung function domain also merits attention. Some definitions require stability, others mandate FEV_1_ ≥ 80% predicted, or either. In our analyses, we used the cut-off of pre-bronchodilator FEV_1_ ≥ 80% predicted, corresponding to normal lung function. This choice was made because, over an observation period of up to 4 years, it is challenging to define “stability” solely in terms of absolute (mL) or relative (%) changes, particularly given the expected age-related decline in lung function. Thus, requiring sustained normal lung function was considered methodologically robust. However, this more stringent definition may have yielded conservative remission estimates. Further studies are needed to establish evidence-based criteria, including whether age-adjusted reference equations are sufficient or additional measures are required.^27^

We could not assess in this analysis whether weight reduction during follow-up— through OCS tapering or lifestyle change —may have contributed to the observed treatment responses.^28^ Nonetheless, this remains an important area for future investigation. Emerging data suggest intentional weight loss can improve asthma control, reduce systemic inflammation, and potentially enhance biologic efficacy.^29^ In addition, weight loss may help mitigate obesity-related comorbidities such as OSAS and GERD, thereby reducing their negative impact on asthma management.^9^

Several limitations should be acknowledged. The observational nature of the registry precludes definitive conclusions regarding causality. BMI was used as the sole measure of obesity, limiting the ability to distinguish between different obesity phenotypes or account for factors such as body composition and metabolic status. Furthermore, information on early-life weight trajectories and on the distinction between steroid-induced and primary obesity was not available in our registry. This lack of mechanistic differentiation precludes exploration of potentially distinct pathophysiological pathways that may influence response to biologic therapy. Peripheral biomarkers such as blood eosinophils and FeNO were available, but more specific assessments of airway inflammation were lacking, which could have provided further insights into disease mechanisms. The analysis of remission was based on a smaller sample and may therefore be underpowered to detect subtle between-group differences. Still, the estimates were consistently close to unity with overlapping confidence intervals, suggesting that any undetected differences are unlikely to materially influence remission trajectories over time. Furthermore, the sensitivity analysis based on multiple imputation, which increased the effective statistical power by incorporating data from all available patients, yielded results that were fully consistent with the primary model (Appendix p 35). Ninety-four patients who had discontinued biologic therapy before baseline were excluded—consistent with our focus on longitudinal outcomes under ongoing treatment—and this exclusion may have introduced attrition-related bias (Appendix p 4), although the proportions of obese and non-obese individuals among early discontinuers closely resembled those of the on-treatment cohort (83% non-obese and 17% obese). The exclusion of a subset of registered patients with missing baseline BMI (n = 129) may have reduced statistical power and introduced bias, as non-random recording of BMI in routine practice cannot be ruled out (Appendix p 4). The increase in 12-month discontinuation (33% vs. 29%), although modest and not statistically significant, together with the slightly shorter follow-up (1%) among naïve patients with BMI ≥ 30 kg/m^2^, does not allow us to fully rule out a differential attrition pattern, despite comparable dropout proportions and reasons across BMI groups (Appendix p 27). Only one death was recorded in our cohort, but literature-based mortality estimates for severe asthma—mostly derived from pre-biologic eras—would suggest higher expected rates than those observed here.^30^ However, the marked reductions in exacerbations, hospitalisations, and emergency visits after biologic initiation, consistent with what has been observed across the broader biologic literature,^1^^,^^3^^,^^9^ suggest that mortality in this population might be considerably lower than in historical cohorts.^30^ Therefore, while some underestimation is possible, we believe that a meaningful competing-risk effect for the survival analyses is unlikely to materially affect the results. Finally, the low number of patients receiving dupilumab or tezepelumab limits conclusions for these therapies.

Nonetheless, the real-world setting, heterogeneous population, and longitudinal design strengthen the generalizability of our findings. The evaluation of outcomes at multiple time points, with up to 4 years of follow-up, and the inclusion of both biologic-naïve and on-treatment patients provided valuable insight into the temporal evolution of disease control, lung function, and remission, capturing both long-term and delayed remission dynamics and offering a more nuanced perspective than previous studies.^10^, ^11^, ^12^, ^13^ Comparative analyses between obese and non-obese patients were consistent across all major endpoints, including ACT, AQLQ, FEV_1_, exacerbations, and remission. This coherence across diverse outcome domains reinforces the robustness of our findings and reduces the likelihood that the observed equivalence between groups is attributable to chance or to endpoint-specific variability. A further strength of this study is the extensive sensitivity analysis performed for BMI, including modelling BMI as a continuous variable using both linear and non-linear approaches, as well as stratification into three BMI categories. These analyses suggested a potential, statistically non-significant trend toward better outcomes in normal-weight patients compared with those who were overweight or obese (Appendix p 22, and p 32), with differences between the overweight and obese groups appearing somewhat less pronounced. Finally, the study draws on data from over 60 specialized asthma centres across Italy, providing broad geographic and clinical representation, with no significant between-centre variation as confirmed by sensitivity analyses to account for clustering within centres. Although the cohort originates from a single country with a relatively homogeneous ethnic background, the multicentre design enhances the external validity of our findings and supports their applicability in comparable healthcare contexts.

Taken together, these findings provide evidence that biologic agents remain efficacious in obese patients with type-2 high severe asthma, regardless of baseline BMI. Clinical outcomes—including exacerbation rates, asthma control, lung function, and remission rates—improved consistently across BMI categories over time. Concurrent weight-management interventions—including dietary modification, regular exercise, and, when appropriate, pharmacotherapy such as GLP-1 receptor agonists—may further enhance remission rates.^9^ Future investigations should also explore whether specific obesity phenotypes, beyond BMI-defined categories, influence treatment response, and whether metabolic–inflammatory profiling may help refine prognostic assessment and guide more personalised management approaches.

## Contributors

Giorgio Walter Canonica, Danilo Di Bona, and Roberta Parente conceived the study.

Giuseppe Di Gioia and Danilo Di Bona performed the data analysis. Danilo Di Bona drafted the manuscript, which was critically revised by Enrico Heffler, Francesco Blasi, Giorgio Walter Canonica, Roberta Parente, Giuseppe Di Gioia, Gaetano Serviddio, and Pierluigi Paggiaro.

Danilo Di Bona and Giuseppe Di Gioia had direct access to and verified the underlying data.

All authors approved the final version of the manuscript and were responsible for the decision to submit it. There were no academic–commercial partnerships involved in this study.

## Data sharing statement

The data analysed in this study were obtained from the Severe Asthma Network Italy (SANI) registry and are not publicly available due to ethical, legal, and data protection restrictions. De-identified individual participant data may be made available upon reasonable request to the SANI Steering Committee, subject to approval of a methodologically sound proposal and in accordance with applicable data protection regulations.

## Declaration of generative AI and AI-assisted technologies in the writing process

DDB used ChatGPT (OpenAI, San Francisco, CA, USA) to improve readability and correct grammatical errors in the manuscript. The authors reviewed and approved all AI-assisted edits and take full responsibility for the content of the publication.

## Declaration of interests

DDB reports consultancy fees from Stallergenes-Greer.

EH received personal fees for advisory boards participation and/o speaker's fee from: Sanofi, Regeneron, Astrazeneca, GSK, Celltrion Healthcare, Apogee Therapeutics, Chiesi, Lofarma, Allergy Therapeutics, Almirall, Recordati, Orion Pharma, Firma, Gentili, Blueprint Medicines, Bosch Healtcare, outside the submitted work.

FB has received grants from AstraZeneca, Chiesi Farmaceutici and Insmed, and has received consulting fees from Menarini; F. Blasi also reports payment/honoraria for lectures and advisory boards received from AstraZeneca, Boehringer Ingelheim, Chiesi Farmaceutici, GlaxoSmithKline, Grifols, Insmed, Menarini, Novartis, OM Pharma, Pfizer, Sanofi, Vertex Pharmaceuticals, and Zambon outside the submitted work.

GWC reports having received research grants as well as being lecturer or having received advisory board fees from: Menarini, Anallergo, Allergy Therapeutics, AstraZeneca, Celltrion, Chiesi Farmaceutici, Faes, Firma, Genentech, Guidotti Glaxo Smith Kline, Hal Allergy, Innovacaremd, Novartis, OmPharma, RedMaple, Sanofi-Aventis, Sanofi-Genzyme, Stallergenes-Greer, Uriach Pharma.

GDG, PP, RP, and GS, declare no competing interests.

## Appendix A Supplementary data

Appendix_LRM_R3_clean

SANI network
