# Supplementary material for: Clinical outcomes and remission trajectories in obese and non-obese patients with severe asthma treated with biologics: a retrospective longitudinal cohort study from the Severe Asthma Network Italy (SANI) registry
Source: Lancet Reg Health Eur. 2026 May 7;66:101695. doi: 10.1016/j.lanepe.2026.101695 (PMC13186060; doi:10.1016/j.lanepe.2026.101695)
Supplement: Appendix_LRM_R3_clean [file mmc1.docx]

**Online Supplement**

**Table of Contents**

Online supplement 1: National reimbursement criteria for biologic therapy. p 2

Online supplement 2: **Definitions of Remission (modified from the SANI Expert Consensus) p 3**

Online supplement 3: **Definition of T2-High Profile p 3**

Online supplement 4: Flow-Chart p 4

Online supplement 5: Days on biologic therapy at baseline for each agent, according to BMI category p 5

Online supplement 6. Distribution of Biologic Therapies at Inclusion According to BMI and Treatment

Status (Biologic-Naïve vs. On Biologics) p 6

Online supplement 7. Completeness of Baseline Data Among Biologic-naïve Patients at Inclusion, by

BMI Category p 7

Online supplement 8. Completeness of Baseline Data Among on-biologics Patients at Inclusion, by

BMI Category p 9

Online supplement 9. ZINB-estimated IRR for exacerbations (A), emergency department visits (B),

hospitalizations (C), and unscheduled visits (D), stratified by BMI and

treatment group p 11

Online supplement 10. Estimated number of events for exacerbations, emergency department visits,

hospitalizations, and unscheduled visits, stratified by BMI and treatment group. p 13

Online supplement 11. Linear mixed-effects models-estimated β changes for ACT (A), FEV1 % (B),

AQLQ (C), stratified by BMI and treatment group. p 15

Online supplement 12: Predicted rate of asthma exacerbations over time by treatment group and

BMI category. p 17

Online supplement 13. ZINB-estimated IRR for exacerbations (A), emergency department visits (B),

hospitalizations (C), and unscheduled visits (D), stratified by BMI and

treatment group p 18

Online supplement 14: Predicted rate of ACT, AQLQ and FEV_1_% improvement over time by treatment

group and BMI category p 22

Online supplement 15. Linear mixed-effects models-estimated β changes for ACT (A), FEV1 % (B),

AQLQ (C), stratified by BMI and treatment group p 23

Online supplement 16. Baseline Clinical Remission Status According to BMI and Biologic Exposure p 26

Online supplement 17. Dropouts and related adverse events p 27

Online supplement 18. Adverse events p 28

Online supplement 19. Follow-up time according to BMI and Biologic Exposure p 29

Online supplement 20. Cox proportional hazards models estimating hazard ratios for incident partial or

complete remission, stratified by treatment group and accounting for random

effects due to the multicentre study design. p 29

Online supplement 21. Cox proportional hazards models estimating hazard ratios for incident partial or

complete remission, excluding biologic-naïve patients who initiated treatment

within one month prior to enrolment (n=71). p 29

Online supplement 22. Cox model–estimated hazard ratios for incident partial or complete remission,

stratified by treatment group, with the risk period starting at 12 months. p 30

Online supplement 23. Restricted cubic spline–adjusted Cox proportional hazards model for the

association between BMI and complete or partial remission. p 31

Online supplement 24. Partial or complete clinical remission stratified by BMI and treatment status. p 32

Online supplement 25. Partial or complete clinical remission stratified by BMI and smoking status. p 33

Online supplement 26. **Multiple imputation–based sensitivity analyses for Cox proportional hazards**

**models** p 34

Online supplement 27. Cox proportional hazards model estimates and numbers at risk across 20

multiply imputed datasets in naïve and on-biologics patients. p 35

**Online supplement 1: National reimbursement criteria for biologic therapy**

**1. Omalizumab** (All eligibility criteria must be met)

- Severe persistent allergic asthma >12 months, uncontrolled despite maximal inhaled therapy.
- Requirement for systemic corticosteroids (adults/adolescents ≥12 yrs; children 6–12 yrs).
- Sensitization to a perennial allergen (skin test or PRIST/RAST).
- Total serum IgE: ≥76 IU/ml (adolescents/adults) or ≥200 IU/ml (children) **and** positive allergen test.
- FEV1 <80% predicted **and** poor asthma control (ongoing treatment, rescue medication, or hospitalizations).

**2. Mepolizumab**

- Blood eosinophils >300 cells/µl in past 12 months **AND** >150 cells/µl currently (off systemic steroids).
- Plus ≥1 of the following:
  1. ≥2 exacerbations in past 12 months despite maximal inhaled therapy (≥12 yrs) / tolerated therapy (6–11 yrs).
  2. Continuous oral corticosteroids ≥6 months in past year (adults only).

**3. Benralizumab**

- Adults with severe refractory eosinophilic asthma.
- Eosinophils ≥300 cells/µl (off systemic steroids).
- Plus ≥1 of the following:
  1. ≥2 exacerbations in past 12 months despite maximal inhaled therapy (GINA step 4–5).
  2. Continuous oral corticosteroids in addition to maximal inhaled therapy in past year.

**4. Dupilumab**

- Severe asthma with type 2 inflammation (age ≥6 yrs).
- Eosinophils ≥150 cells/µl **OR** FeNO >25 ppm.
- Plus ≥1 of the following:
  1. ≥2 exacerbations in past 12 months despite maximal inhaled therapy.
  2. Continuous oral corticosteroids (≥6 months in adults; ≥1 month in children 6–17 yrs).

**5. Tezepelumab**

**Eligibility Criteria for Tezepelumab (All criteria must be met)**

- Severe uncontrolled asthma (age ≥12 yrs) despite high-dose inhaled corticosteroids.
- In the past 12 months:
  • ≥2 exacerbations despite maximal inhaled therapy, requiring systemic steroids or hospitalization, **AND**
  • Continuous oral corticosteroids (≥6 months in adults; ≥1 month in adolescents).

**Online supplement 2: Definitions of Remission (modified from the SANI Expert Consensus)***

**Definition**
Clinical remission is defined as a composite measure based on multiple criteria. Remission was considered present when the following criteria were concurrently met for a minimum duration of 12 months:

1. No further need for oral corticosteroid treatment
2. Absence of asthma exacerbations or attacks
3. Absence of asthma symptoms (ACT≥20)
4. Stable normal lung function (pre-bronchodilator FEV_1_≥80%).*

**Duration**
All criteria must be met for at least 12 months.

**Assessment Scores**

- Asthma Control Test (ACT): score between 20/25 and 25/25
- Asthma Control Questionnaire (ACQ): score <1·5

**Remission Categories**
Two levels of remission have been defined:

1. **Partial remission**: No further need for oral corticosteroids plus any **two** of the remaining three criteria
2. **Complete remission**: Fulfilment of **all four** criteria

** This modification entails the addition of a pre-bronchodilator FEV₁ ≥ 80% of the predicted value to the criterion of stable lung function, thereby redefining it as “stable normal lung function.*

**Online supplement 3: Definition of T2-High Profile**

T2-high inflammation was defined as the presence of at least two of the following three criteria:

- Fractional exhaled nitric oxide (FeNO) ≥ 25 ppb
- Blood eosinophil count ≥ 300 cells/µL
- Total serum IgE ≥ 100 IU/mL

**Online supplement 4: Flow-Chart**

**SANI registry:**

3193

**No biologic treatment:** 799

**Missing assessment dates:** 54

**Missing baseline data (age, sex):** 18

**Missing baseline BMI:** 129

**Biologic treatment stopped before baseline:** 94

**Study Population:**

2098

**On biologics at inclusion:**

980

**Biologic-naïve at inclusion:**

1118

Flow-chart illustrating the selection of the study population from the SANI registry (n=3·193). Exclusion criteria included absence of biologic treatment, missing assessment dates, missing baseline data (age, sex, BMI), or biologic treatment stopped before baseline. The final study population (n=2·098) was stratified into patients already on biologics at inclusion and biologic-naïve patients. Numbers relating to each subsequent analysis are reported in the corresponding supplementary tables.

**Online supplement 5: Days on biologic therapy at baseline for each agent, according to BMI category.**

|  | **On biologics at inclusion** | | | |
| --- | --- | --- | --- | --- |
|  | **Total** | **BMI<30** | **BMI≥30** | ***P*** |
|  | N=980 | N=800 | N=180 |  |
| **Overall**, mean (SD) | 935·55 (1111·63) | 906·06 (1070·92) | 1066·62 (1272·04) | 0·080 |
|  |  |  |  |  |
| **Benralizumab,** mean (SD) | 395·69 (350·38) | 406·71 (369·28) | 349·69 (257·15) | 0·433 |
| **Dupilumab,** mean (SD) | 355·22 (264·77) | 337·27 (251·95) | 474·89 (330·42) | 0·147 |
| **Mepolizumab,** mean (SD) | 458·81 (419·93) | 453·89 (423·37) | 480·39 (407·09) | 0·652 |
| **Omalizumab,** mean (SD) | 1617·50 (1373·46) | 1559·89 (1352·74) | 1864·71 (1546·75) | 0·076 |
| **Tezepelumab,** mean (SD) | 107 (91·82) | 107 (91·82) | 0 | N/A |

SD, standard deviation; N/A, not available.

Mean (± SD) number of days on biologic therapy at study inclusion for the on biologics group, stratified by drug type and BMI category (<30 vs. ≥30). Reported for each agent (Benralizumab, Dupilumab, Mepolizumab, Omalizumab, Tezepelumab) and overall P values indicate comparisons between BMI categories.

**Online supplement 6. Distribution of Biologic Therapies at Inclusion According to BMI and Treatment Status (Biologic-Naïve vs. On Biologics)**

|  | **Biologic-naïve at inclusion** | | | | **On biologics at inclusion** | | | |
| --- | --- | --- | --- | --- | --- | --- | --- | --- |
|  | **Total** | **BMI<30** | **BMI≥30** | ***P*** | **Total** | **BMI<30** | **BMI≥30** | ***P*** |
|  | N=1118 | N=893 | N=225 |  | N=980 | N=800 | N=180 |  |
| **Overall** | 1110 | 885 | 225 | 0·315 | 979 | 799 | 180 | 0·706 |
|  |  |  |  |  |  |  |  |  |
| **Omalizumab** | 223 | 170 | 53 |  | 418 | 339 | 79 |  |
| **Mepolizumab** | 390 | 310 | 80 |  | 339 | 276 | 63 |  |
| **Benralizumab** | 272 | 226 | 46 |  | 150 | 121 | 29 |  |
| **Dupilumab** | 179 | 145 | 34 |  | 69 | 60 | 9 |  |
| **Tezepelumab** | 46 | 34 | 12 |  | 3 | 3 | 0 |  |

Distribution of biologic agents among patients naïve to biologics and those already on biologic therapy at inclusion, stratified by BMI (<30 vs ≥30). Data are presented as absolute numbers. P values indicate comparisons between BMI categories.

**Online supplement 7. Completeness of Baseline Data Among Biologic-naïve Patients at Inclusion, by BMI Category.**

|  | **Biologic-naïve at inclusion (non-missing data)** | | | **Biologic-naïve at inclusion (missing data)** | | |
| --- | --- | --- | --- | --- | --- | --- |
|  | **Total** | **BMI<30** | **BMI≥30** | **Total** | **BMI<30** | **BMI≥30** |
|  | 1118 | 893 | 225 | 0 | 0 | 0 |
| Age | 1118 | 893 | 225 | 0 | 0 | 0 |
| Sex (Female) | 1118 | 893 | 225 | 0 | 0 | 0 |
| BMI | 1118 | 893 | 225 | 0 | 0 | 0 |
| Smoke (smokers/former) | 1109 | 885 | 224 | 9 | 8 | 1 |
| Onset asthma (age) | 820 | 651 | 169 | 298 | 242 | 56 |
| Diagnosis asthma (age) | 830 | 659 | 171 | 288 | 234 | 54 |
| **Clinical status** |  |  |  |  |  |  |
| ACQ >1·5 or ACT <20 or uncontrolled according to GINA | 922 | 741 | 181 | 196 | 152 | 44 |
| ACT | 989 | 788 | 201 | 129 | 105 | 24 |
| AQLQ | 811 | 654 | 157 | 307 | 239 | 68 |
| FEV1 post-bronchodilator <80% of predicted | 922 | 741 | 181 | 196 | 152 | 44 |
| FEV1 pre-bronchodilator (%) | 819 | 652 | 167 | 299 | 241 | 58 |
| FEV1 post-bronchodilator (%) | 435 | 349 | 86 | 683 | 544 | 139 |
| Patients with ≥2 exacerbations treated with systemic CS >3 consecutive days in the year before biologic | 922 | 741 | 181 | 196 | 152 | 44 |
| Exacerbations  requiring corticosteroids | 1118 | 893 | 225 | 0 | 0 | 0 |
| At least one hospitalization, ICU admission, or mechanical ventilation for asthma in the past year | 922 | 741 | 181 | 196 | 152 | 44 |
| Asthma related hospitalizations | 1118 | 893 | 225 | 0 | 0 | 0 |
| Asthma related ED visits | 1118 | 893 | 225 | 0 | 0 | 0 |
| No scheduled visits | 1118 | 893 | 225 | 0 | 0 | 0 |
| **On treatment with:** |  |  |  |  |  |  |
| - High doses of inhaled corticosteroids plus LABA or another controller | 922 | 741 | 181 | 196 | 152 | 44 |
| - OCS for at least six months in the past year | 922 | 741 | 181 | 196 | 152 | 44 |
| **Average daily prednisone-equivalent dosage in the year before baseline** | 115 | 91 | 24 | 1·003 | 802 | 201 |
| **Biomarkers** |  |  |  |  |  |  |
| FeNO | 550 | 440 | 110 | 568 | 453 | 115 |
| Eosinophils (n) | 950 | 762 | 188 | 168 | 131 | 37 |
| Neutrophils (n) | 693 | 552 | 141 | 425 | 341 | 84 |
| Total IgE | 699 | 561 | 138 | 419 | 332 | 87 |
| Th2 profile | 1118 | 893 | 225 | 0 | 0 | 0 |
| **Comorbidities** |  |  |  |  |  |  |
| Allergy | 1118 | 893 | 225 | 0 | 0 | 0 |
| Rhinitis | 1108 | 886 | 222 | 10 | 7 | 3 |
| CRSsNP | 1096 | 875 | 221 | 22 | 18 | 4 |
| CRSwNP | 1116 | 891 | 225 | 2 | 2 | 0 |
| OSAS | 1052 | 840 | 212 | 66 | 53 | 13 |
| GI reflux | 1099 | 876 | 223 | 19 | 17 | 2 |
| Cardiovascular disease | 1060 | 843 | 217 | 58 | 50 | 8 |
| Type 2 diabetes | 1061 | 846 | 215 | 57 | 47 | 10 |
| Osteoporosis | 963 | 768 | 195 | 155 | 125 | 30 |
| Mood disorders | 1060 | 845 | 215 | 58 | 48 | 10 |

Completeness of baseline data in biologic-naïve patients at inclusion, stratified by BMI (<30 vs. ≥30). For each clinical, functional, biomarker, and comorbidity variable, the table reports the number of patients with available and missing data.

Data are presented as means ± standard deviation (SD), harmonic means (×/multiplicative SD) or number (%).

**Abbreviations**: BMI, body mass index; ACT, asthma control test; ACQ, asthma control questionnaire; GINA, Global Initiative for Asthma; AQLQ, asthma quality of life questionnaire; FEV_1_, forced expiratory volume in 1 second; CS, corticosteroid; ICU, intensive care unit; ED, emergency department; LABA, long-acting beta agonist; OCS, oral corticosteroids; FeNO, Fractional Exhaled Nitric Oxide; IgE, immunoglobulin E; CRSsNP, chronic rhinosinusitis without Nasal Polyps; CRSwNP, chronic rhinosinusitis with Nasal Polyps; OSAS, obstructive sleep apnoea syndrome; GI, gastrointestinal.

**Online supplement 8. Completeness of Baseline Data Among on-biologics Patients at Inclusion, by BMI Category.**

|  | **On biologics at inclusion (non-missing data)** | | | **On biologics at inclusion (missing data)** | | |
| --- | --- | --- | --- | --- | --- | --- |
|  | **Total** | **BMI<30** | **BMI≥30** | **Total** | **BMI<30** | **BMI≥30** |
|  | N=980 | N=800 | N=180 | N=0 | N=0 | N=0 |
| Age | 980 | 800 | 180 | 0 | 0 | 0 |
| Sex (Female) | 980 | 800 | 180 | 0 | 0 | 0 |
| BMI | 980 | 800 | 180 | 0 | 0 | 0 |
| Smoke (smokers/former) | 980 | 800 | 180 | 0 | 0 | 0 |
| Onset asthma (age) | 969 | 794 | 175 | 11 | 6 | 5 |
| Diagnosis asthma (age) | 879 | 723 | 156 | 101 | 77 | 24 |
| **Clinical status** |  |  |  |  |  |  |
| ACQ >1·5 or ACT <20 or uncontrolled according to GINA | 980 | 800 | 180 | 0 | 0 | 0 |
| ACT | 869 | 715 | 154 | 111 | 85 | 26 |
| AQLQ | 653 | 537 | 116 | 327 | 263 | 64 |
| FEV1 post-bronchodilator <80% of predicted | 980 | 800 | 180 | 0 | 0 | 0 |
| FEV1 pre-bronchodilator (%) | 707 | 576 | 131 | 273 | 224 | 49 |
| FEV1 post-bronchodilator (%) | 337 | 271 | 66 | 643 | 529 | 114 |
| Patients with ≥2 exacerbations treated with systemic CS >3 consecutive days in the year before biologic | 980 | 800 | 180 | 0 | 0 | 0 |
| Exacerbations  requiring corticosteroids | 980 | 800 | 180 | 0 | 0 | 0 |
| At least one hospitalization, ICU admission, or mechanical ventilation for asthma in the past year | 980 | 800 | 180 | 0 | 0 | 0 |
| Asthma related hospitalizations | 980 | 800 | 180 | 0 | 0 | 0 |
| Asthma related ED visits | 980 | 800 | 180 | 0 | 0 | 0 |
| No scheduled visits | 980 | 800 | 180 | 0 | 0 | 0 |
| **On treatment with:** |  |  |  |  |  |  |
| - High doses of inhaled corticosteroids plus LABA or another controller | 980 | 800 | 180 | 0 | 0 | 0 |
| - OCS for at least six months in the past year | 980 | 800 | 180 | 0 | 0 | 0 |
| **Average daily prednisone-equivalent dosage in the year before baseline** | 63 | 46 | 17 | 917 | 754 | 163 |
| **Biomarkers** |  |  |  |  |  |  |
| FeNO |  |  |  |  |  |  |
| Eosinophils (n) | 443 | 373 | 70 | 537 | 427 | 110 |
| Neutrophils (n) | 730 | 603 | 127 | 250 | 197 | 53 |
| Total IgE | 537 | 447 | 90 | 443 | 353 | 90 |
| Th2 profile | 555 | 467 | 88 | 425 | 333 | 92 |
| **Comorbidities** |  |  |  |  |  |  |
| Allergy | 980 | 800 | 180 | 0 | 0 | 0 |
| Rhinitis | 966 | 792 | 174 | 14 | 8 | 6 |
| CRSsNP | 962 | 790 | 172 | 18 | 10 | 8 |
| CRSwNP | 970 | 795 | 175 | 10 | 5 | 5 |
| OSAS | 934 | 766 | 168 | 46 | 34 | 12 |
| GI reflux | 966 | 792 | 174 | 14 | 8 | 6 |
| Cardiovascular disease | 914 | 749 | 165 | 66 | 51 | 15 |
| Type 2 diabetes | 918 | 749 | 169 | 62 | 51 | 11 |
| Osteoporosis | 858 | 708 | 150 | 122 | 92 | 30 |
| Mood disorders | 916 | 747 | 169 | 64 | 53 | 11 |

Completeness of baseline data in patients already on biologics at inclusion, stratified by BMI (<30 vs. ≥30). The table reports the number of patients with available and missing data for each clinical, functional, biomarker, and comorbidity variable.

Data are presented as means ± standard deviation (SD), harmonic means (×/multiplicative SD) or number (%).

**Abbreviations**: BMI, body mass index; ACT, asthma control test; ACQ, asthma control questionnaire; GINA, Global Initiative for Asthma; AQLQ, asthma quality of life questionnaire; FEV_1_, forced expiratory volume in 1 second; CS, corticosteroid; ICU, intensive care unit; ED, emergency department; LABA, long-acting beta agonist; OCS, oral corticosteroids; FeNO, Fractional Exhaled Nitric Oxide; IgE, immunoglobulin E; CRSsNP, chronic rhinosinusitis without Nasal Polyps; CRSwNP, chronic rhinosinusitis with Nasal Polyps; OSAS, obstructive sleep apnoea syndrome; GI, gastrointestinal.

# **Online supplement 9. ZINB-estimated IRR for exacerbations (A), emergency department visits (B), hospitalizations (C), and unscheduled visits (D), stratified by BMI and treatment group.**

**A. Exacerbations requiring systemic corticosteroid**

|  | **Naïve** | | | | **On biologics** | | | |
| --- | --- | --- | --- | --- | --- | --- | --- | --- |
| **Time (months)** |  | **BMI<30** |  | **BMI≥30** |  | **BMI<30** |  | **BMI≥30** |
|  | **n** | **IRR (95% CI)** | **n** | **IRR (95% CI)** | **n** | **IRR (95% CI)** | **n** | **IRR (95% CI)** |
| 0 | 665 | Reference | 166 | 0·87 (0·68; 1·06) | 591 | 0·65 (0·52; 0·77) | 133 | 0·80 (0·42; 1·19) |
| 6 | 607 | 0·33 (0·28; 0·38) | 143 | 0·35 (0·26; 0·45) | 547 | 0·23 (0·19; 0·27) | 119 | 0·25 (0·13; 0·37) |
| 12 | 488 | 0·11 (0·09; 0·14) | 117 | 0·14 (0·09; 0·20) | 439 | 0·08 (0·06; 0·10) | 92 | 0·08 (0·01; 0·14) |
| 18 | 404 | 0·04 (0·03; 0·05) | 94 | 0·06 (0·03; 0·09) | 369 | 0·03 (0·02; 0·04) | 79 | 0·03 (0·00; 0·06) |
| 24 | 335 | 0·01 (0·01; 0·02) | 81 | 0·02 (0·01; 0·04) | 314 | 0·01 (0·01; 0·01) | 70 | 0·01 (0·00; 0·02) |

*Multi-adjusted for baseline age, sex,* CRSwNP*, baseline eosinophils (n), baseline FEV1, baseline ACT, days on biologic therapy at baseline.*

**B. Emergency Department visits**

|  | **Naïve** | | | | **On biologics** | | | |
| --- | --- | --- | --- | --- | --- | --- | --- | --- |
| **Time (months)** |  | **BMI<30** |  | **BMI≥30** |  | **BMI<30** |  | **BMI≥30** |
|  | **n** | **IRR (95% CI)** | **n** | **IRR (95% CI)** | **n** | **IRR (95% CI)** | **n** | **IRR (95% CI)** |
| 0 | 681 | Reference | 169 | 0·90 (0·56; 1·25) | 588 | 0·82 (0·51; 1·12) | 130 | 1·22 (0·27; 2·17) |
| 6 | 609 | 0·38 (0·29; 0·47) | 144 | 0·41 (0·26; 0·56) | 552 | 0·30 (0·18; 0·42) | 121 | 0·41 (0·13; 0·69) |
| 12 | 489 | 0·14 (0·10; 0·18) | 117 | 0·19 (0·11; 0·26) | 440 | 0·11 (0·05; 0·17) | 92 | 0·14 (0·04; 0·24) |
| 18 | 405 | 0·05 (0·04; 0·07) | 94 | 0·08 (0·04; 0·12) | 370 | 0·04 (0·01; 0·07) | 79 | 0·05 (0·01; 0·09) |
| 24 | 337 | 0·02 (0·01; 0·03) | 81 | 0·04 (0·02; 0·06) | 314 | 0·01 (0·00; 0·03) | 70 | 0·02 (0·00; 0·03) |

*Multi-adjusted for baseline age, sex,* CRSwNP*, baseline eosinophils (n), baseline FEV1, baseline ACT, days on biologic therapy at baseline.*

**C. Hospitalizations**

|  | **Naïve** | | | | **On biologics** | | | |
| --- | --- | --- | --- | --- | --- | --- | --- | --- |
| **Time (months)** |  | **BMI<30** |  | **BMI≥30** |  | **BMI<30** |  | **BMI≥30** |
|  | **n** | **IRR (95% CI)** | **n** | **IRR (95% CI)** | **n** | **IRR (95% CI)** | **n** | **IRR (95% CI)** |
| 0 | 686 | Reference | 170 | 0·65 (0·33; 0·96) | 600 | 1·03 (0·63; 1·42) | 137 | 0·85 (0·21; 1·49) |
| 6 | 609 | 0·16 (0·00; 0·36) | 143 | 0·28 (0·11; 0·45) | 552 | 0·31 (0·16; 0·47) | 121 | 0·25 (0·00; 0·63) |
| 12 | 489 | 0·03 (0·00; 0·09) | 117 | 0·12 (0·02; 0·22) | 440 | 0·10 (0·01; 0·18) | 92 | 0·07 (0·00; 0·27) |
| 18 | 405 | 0·00 (0·00; 0·02) | 94 | 0·05 (0·00; 0·11) | 370 | 0·03 (0·00; 0·07) | 79 | 0·02 (0·00; 0·11) |
| 24 | 337 | 0·00 (0·00; 0·00) | 81 | 0·02 (0·00; 0·06) | 314 | 0·01 (0·00; 0·02) | 70 | 0·01 (0·00; 0·04) |

*Multi-adjusted for baseline age, sex,* CRSwNP*, baseline eosinophils (n), baseline FEV1, baseline ACT, days on biologic therapy at baseline.*

**D. Unscheduled visits**

|  | **Naïve** | | | | **On biologics** | | | |
| --- | --- | --- | --- | --- | --- | --- | --- | --- |
| **Time (months)** |  | **BMI<30** |  | **BMI≥30** |  | **BMI<30** |  | **BMI≥30** |
|  | **n** | **IRR (95% CI)** | **n** | **IRR (95% CI)** | **n** | **IRR (95% CI)** | **n** | **IRR (95% CI)** |
| 0 | 556 | Reference | 140 | 0·76 (0·53; 0·99) | 547 | 0·72 (0·54; 0·91) | 113 | 0·60 (0·25; 0·94) |
| 6 | 519 | 0·19 (0·13; 0·25) | 134 | 0·16 (0·06; 0·26) | 540 | 0·22 (0·15; 0·28) | 104 | 0·15 (0·06; 0·24) |
| 12 | 453 | 0·04 (0·01; 0·06) | 108 | 0·04 (0·00; 0·08) | 432 | 0·07 (0·04; 0·10) | 88 | 0·04 (0·01; 0·07) |
| 18 | 378 | 0·01 (0·00; 0·01) | 87 | 0·01 (0·00; 0·02) | 366 | 0·02 (0·01; 0·03) | 77 | 0·01 (0·00; 0·02) |
| 24 | 314 | 0·00 (0·00; 0·00) | 78 | 0·00 (0·00; 0·01) | 308 | 0·01 (0·00; 0·01) | 69 | 0·00 (0·00; 0·01) |

*Multi-adjusted for baseline age, sex,* CRSwNP*, baseline eosinophils (n), baseline FEV1, baseline ACT, days on biologic therapy at baseline.*

Zero-inflated negative binomial (ZINB) regression models estimating incidence rate ratios (IRR) for exacerbations requiring systemic corticosteroids, emergency department visits, hospitalizations, and unscheduled visits. Results are stratified by BMI category (<30 vs. ≥30), treatment status (biologic-naïve vs. on biologics), and follow-up time (0–24 months). Models are adjusted for baseline age, sex, CRSwNP, eosinophils, FEV1, ACT, and days on biologic therapy at inclusion. The reference category is the biologic-naïve group at baseline (T=0), which allows IRR comparisons both within the naïve population over time and between naïve and on-treatment patients.

**Online supplement 10. Estimated number of events for exacerbations, emergency department visits, hospitalizations, and unscheduled visits, stratified by BMI and treatment group.**

|  | |  | **Exacerbations** | **ED visits** | **Hospitalizations** | **Unscheduled visits** |
| --- | --- | --- | --- | --- | --- | --- |
| **Group** | **Time (months)** | | **Events (95%CI)** | **Events (95%CI)** | **Events (95%CI)** | **Events (95%CI)** |
| Naive - BMI<30 | 0 | | 8·33 (7·35, 9·31) | 0·7 (0·53, 0·86) | 0·48 (0·32, 0·63) | 3·0 (2·47, 3·54) |
| Naive - BMI≥ 30 | 0 | | 7·27 (5·68, 8·86) | 0·63 (0·39, 0·87) | 0·31 (0·16, 0·46) | 2·27 (1·58, 2·96) |
| On biologics - BMI<30 | 0 | | 5·39 (4·35, 6·42) | 0·57 (0·36, 0·78) | 0·49 (0·3, 0·68) | 2·17 (1·61, 2·73) |
| On biologics - BMI≥30 | 0 | | 6·69 (3·48, 9·9) | 0·85 (0·19, 1·51) | 0·41 (0·1, 0·72) | 1·79 (0·76, 2·82) |
| Naive - BMI<30 | 6 | | 2·77 (2·37, 3·18) | 0·26 (0·2, 0·32) | 0·08 (0·0, 0·17) | 0·58 (0·4, 0·76) |
| Naive - BMI≥30 | 6 | | 2·94 (2·16, 3·71) | 0·29 (0·18, 0·39) | 0·13 (0·05, 0·21) | 0·49 (0·19, 0·79) |
| On biologics - BMI<30 | 6 | | 1·91 (1·57, 2·26) | 0·21 (0·13, 0·29) | 0·15 (0·08, 0·23) | 0·65 (0·46, 0·85) |
| On biologics - BMI≥30 | 6 | | 2·09 (1·07, 3·12) | 0·29 (0·09, 0·48) | 0·12 (0·0, 0·3) | 0·46 (0·18, 0·73) |
| Naive - BMI<30 | 12 | | 0·92 (0·71, 1·14) | 0·1 (0·07, 0·13) | 0·01 (0·0, 0·04) | 0·11 (0·04, 0·18) |
| Naive - BMI≥30 | 12 | | 1·19 (0·75, 1·63) | 0·13 (0·08, 0·18) | 0·06 (0·01, 0·11) | 0·11 (0·0, 0·23) |
| On biologics - BMI<30 | 12 | | 0·68 (0·53, 0·83) | 0·08 (0·03, 0·12) | 0·05 (0·01, 0·09) | 0·2 (0·11, 0·29) |
| On biologics - BMI≥30 | 12 | | 0·65 (0·12, 1·19) | 0·1 (0·03, 0·17) | 0·03 (0·0, 0·13) | 0·12 (0·01, 0·22) |
| Naive - BMI<30 | 18 | | 0·31 (0·21, 0·41) | 0·04 (0·03, 0·05) | 0·0 (0·0, 0·01) | 0·02 (0·0, 0·04) |
| Naive - BMI≥30 | 18 | | 0·48 (0·24, 0·72) | 0·06 (0·03, 0·09) | 0·02 (0·0, 0·05) | 0·02 (0·0, 0·06) |
| On biologics - BMI<30 | 18 | | 0·24 (0·17, 0·31) | 0·03 (0·01, 0·05) | 0·01 (0·0, 0·03) | 0·06 (0·02, 0·1) |
| On biologics - BMI≥30 | 18 | | 0·2 (0·0, 0·46) | 0·03 (0·01, 0·06) | 0·01 (0·0, 0·05) | 0·03 (0·0, 0·07) |
| Naive - BMI<30 | 24 | | 0·1 (0·06, 0·15) | 0·01 (0·01, 0·02) | 0·0 (0·0, 0·0) | 0·0 (0·0, 0·01) |
| Naive - BMI≥30 | 24 | | 0·19 (0·07, 0·32) | 0·03 (0·01, 0·04) | 0·01 (0·0, 0·03) | 0·0 (0·0, 0·02) |
| On biologics - BMI<30 | 24 | | 0·09 (0·05, 0·12) | 0·01 (0·0, 0·02) | 0·0 (0·0, 0·01) | 0·02 (0·0, 0·03) |
| On biologics - BMI≥30 | 24 | | 0·06 (0·0, 0·17) | 0·01 (0·0, 0·02) | 0·0 (0·0, 0·02) | 0·01 (0·0, 0·02) |

Multi-adjusted for baseline age, sex, CRSwNP, baseline eosinophils (n), baseline FEV1, baseline ACT, days on biologic therapy at baseline. Model-based estimated number of events for exacerbations, emergency department visits, hospitalizations, and unscheduled visits, stratified by BMI category and treatment status, at different follow-up time points (0, 6, 12, 18, 24 months). Data are expressed as mean values with 95% confidence intervals.

**Online supplement 11. Linear mixed-effects models-estimated β changes for ACT (A), FEV1 % (B), AQLQ (C), stratified by BMI and treatment group.**

**A. ACT**

|  | **Naïve** | | | | **On biologics** | | | |
| --- | --- | --- | --- | --- | --- | --- | --- | --- |
| **Time (months)** |  | **BMI<30** |  | **BMI≥30** |  | **BMI<30** |  | **BMI≥30** |
|  | **n** | **β (95% CI)** | **n** | **β (95% CI)** | **n** | **β (95% CI)** | **n** | **β (95% CI)** |
| 0 | 639 | Reference | 162 | -1·63 (-2·41, -0·86) | 573 | Reference | 126 | -0·64 (-1·69, 0·41) |
| 6 | 588 | 0·45 (0·38, 0·51) | 144 | -1·08 (-1·81, -0·36) | 523 | 0·17 (0·11, 0·24) | 111 | -0·41 (-1·40, 0·58) |
| 12 | 469 | 0·89 (0·76, 1·02) | 115 | -0·53 (-1·23, 0·17) | 418 | 0·34 (0·21, 0·47) | 85 | -0·18 (-1·13, 0·78) |
| 18 | 389 | 1·34 (1·15, 1·53) | 91 | 0·02 (-0·68, 0·72) | 350 | 0·51 (0·32, 0·71) | 72 | 0·06 (-0·88, 0·99) |
| 24 | 317 | 1·79 (1·53, 2·04) | 75 | 0·57 (-0·16, 1·29) | 298 | 0·69 (0·42, 0·95) | 62 | 0·29 (-0·66, 1·24) |

*Multi-adjusted for baseline age, sex,* CRSwNP*, baseline eosinophils (n), baseline FEV1, baseline number of exacerbations (n), days on biologic therapy at baseline.*

**B. FEV1%**

|  | **Naïve** | | | | **On biologics** | | | |
| --- | --- | --- | --- | --- | --- | --- | --- | --- |
| **Time (months)** |  | **BMI<30** |  | **BMI≥30** |  | **BMI<30** |  | **BMI≥30** |
|  | **n** | **β (95% CI)** | **n** | **β (95% CI)** | **n** | **β (95% CI)** | **n** | **β (95% CI)** |
| 0 | 546 | Reference | 138 | 0·81 (-3·48, 5·09) | 456 | Reference | 107 | 0·42 (-4·48, 5·33) |
| 6 | 538 | 1·13 (0·85, 1·42) | 126 | 1·48 (-2·69, 5·65) | 409 | 0·31 (0·03, 0·59) | 103 | 0·57 (-4·21, 5·35) |
| 12 | 426 | 2·27 (1·69, 2·85) | 101 | 2·16 (-1·98, 6·30) | 365 | 0·62 (0·06, 1·18) | 81 | 0·72 (-4·04, 5·47) |
| 18 | 350 | 3·40 (2·54, 4·27) | 85 | 2·83 (-1·37, 7·04) | 308 | 0·93 (0·09, 1·77) | 60 | 0·86 (-3·96, 5·68) |
| 24 | 281 | 4·54 (3·39, 5·69) | 76 | 3·51 (-0·84, 7·86) | 261 | 1·24 (0·12, 2·36) | 53 | 1·01 (-3·96, 5·98) |

*Multi-adjusted for baseline age, sex,* CRSwNP*, baseline eosinophilic (n), baseline ACT, baseline number of exacerbations (n), days on biologic therapy at baseline.*

**C. AQLQ**

|  | **Naïve** | | | | **On biologics** | | | |
| --- | --- | --- | --- | --- | --- | --- | --- | --- |
| **Time (months)** |  | **BMI<30** |  | **BMI≥30** |  | **BMI<30** |  | **BMI≥30** |
|  | **n** | **β (95% CI)** | **n** | **β (95% CI)** | **n** | **β (95% CI)** | **n** | **β (95% CI)** |
| 0 | 545 | Ref. | 130 | -0·19 (-0·42, 0·04) | 431 | Ref. | 97 | -0·11 (-0·38, 0·16) |
| 6 | 472 | 0·13 (0·11, 0·15) | 112 | -0·05 (-0·27, 0·16) | 380 | 0·08 (0·06, 0·09) | 79 | 0·00 (-0·25, 0·26) |
| 12 | 378 | 0·25 (0·22, 0·29) | 92 | 0·09 (-0·13, 0·30) | 305 | 0·15 (0·11, 0·19) | 64 | 0·12 (-0·13, 0·37) |
| 18 | 310 | 0·38 (0·32, 0·44) | 76 | 0·22 (0·01, 0·44) | 260 | 0·23 (0·17, 0·28) | 55 | 0·23 (-0·03, 0·49) |
| 24 | 253 | 0·51 (0·43, 0·58) | 63 | 0·36 (0·13, 0·59) | 220 | 0·30 (0·22, 0·38) | 50 | 0·35 (0·08, 0·62) |

*Multi-adjusted for baseline age, sex,* CRSwNP*, baseline eosinophils (n), baseline FEV1, baseline ACT, , baseline number of exacerbations (n), days on biologic therapy at baseline.*

Linear mixed-effects model estimates (β with 95% CI) for changes in ACT, FEV1% predicted, and AQLQ, stratified by treatment status (biologic-naïve vs. on biologics) and BMI category (<30 vs. ≥30) across follow-up (0–24 months). Models are adjusted for baseline age, sex, CRSwNP, eosinophils, FEV1, ACT, number of exacerbations, and days on biologic therapy at inclusion.

**Online supplement 12. Predicted rate of asthma exacerbations over time by treatment group and BMI category.** IRRs for exacerbations requiring corticosteroids, emergency department visits, hospitalisations, and unscheduled visits are shown according to treatment status (biologic-naïve vs on-biologic). Estimates were obtained from zero-inflated negative binomial (ZINB) regression adjusted for baseline age, sex, chronic rhinosinusitis with nasal polyps (CRSwNP), blood eosinophil count, FEV₁ (% predicted), ACT score, and days on biologic therapy at baseline. BMI was modelled as a continuous covariate; for illustrative purposes, predicted curves are displayed for representative BMI values (20, 25, 30, 35, and 40 kg/m²). The reference treatment category is biologic-naïve with BMI=25 at baseline.

**
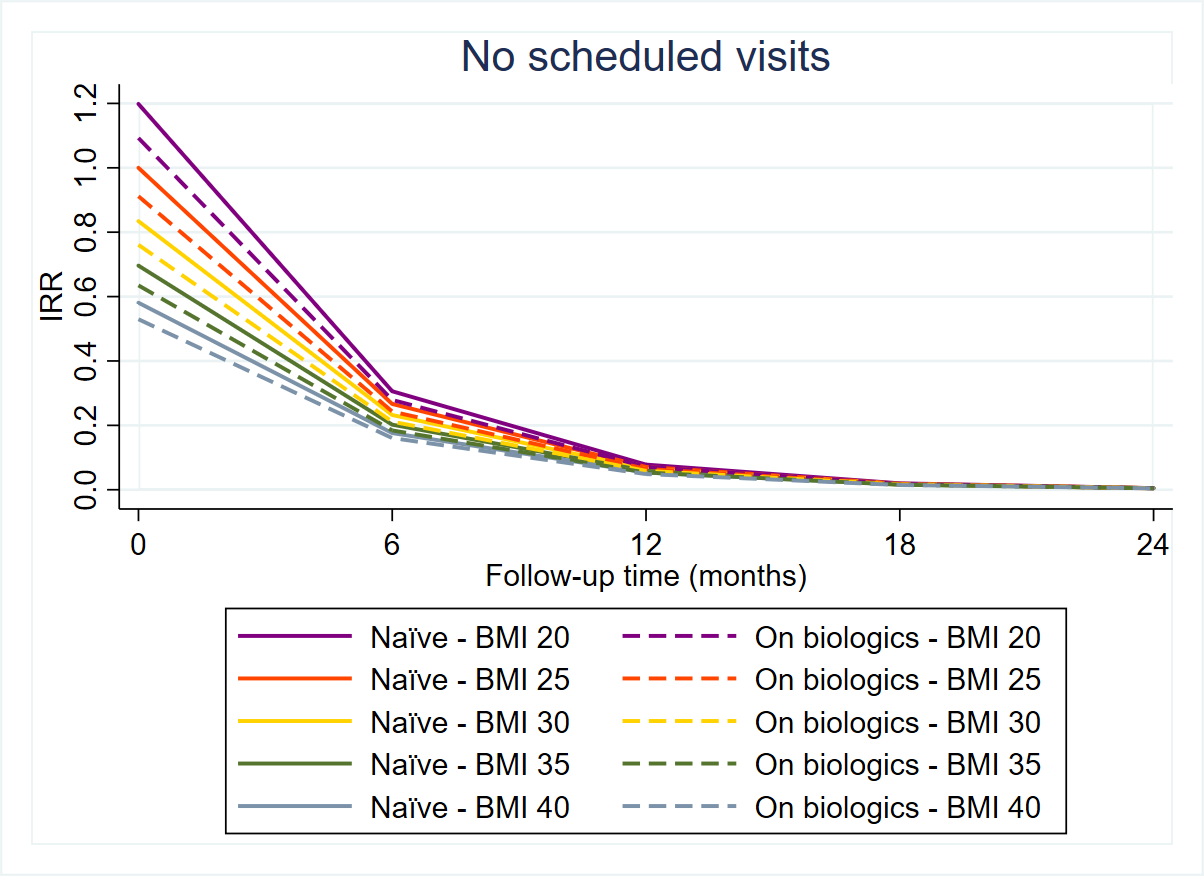

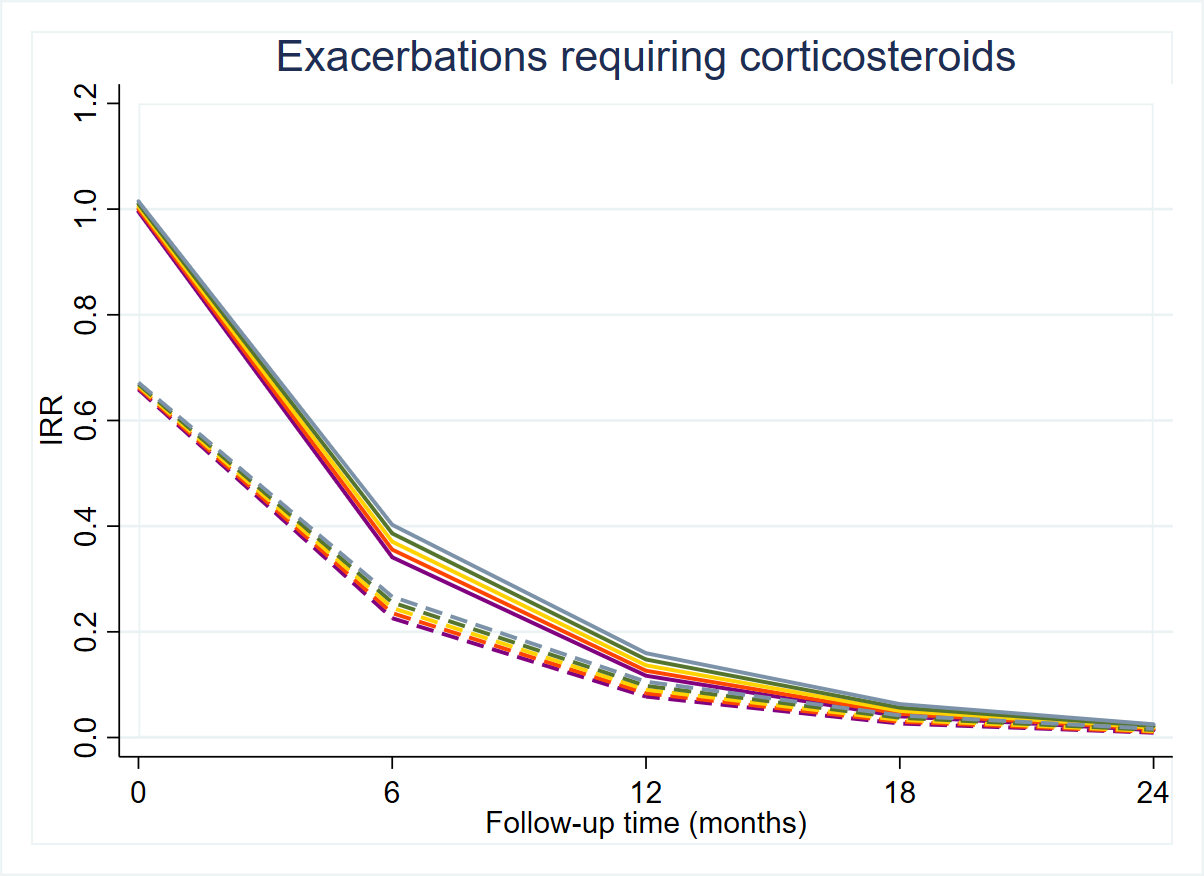

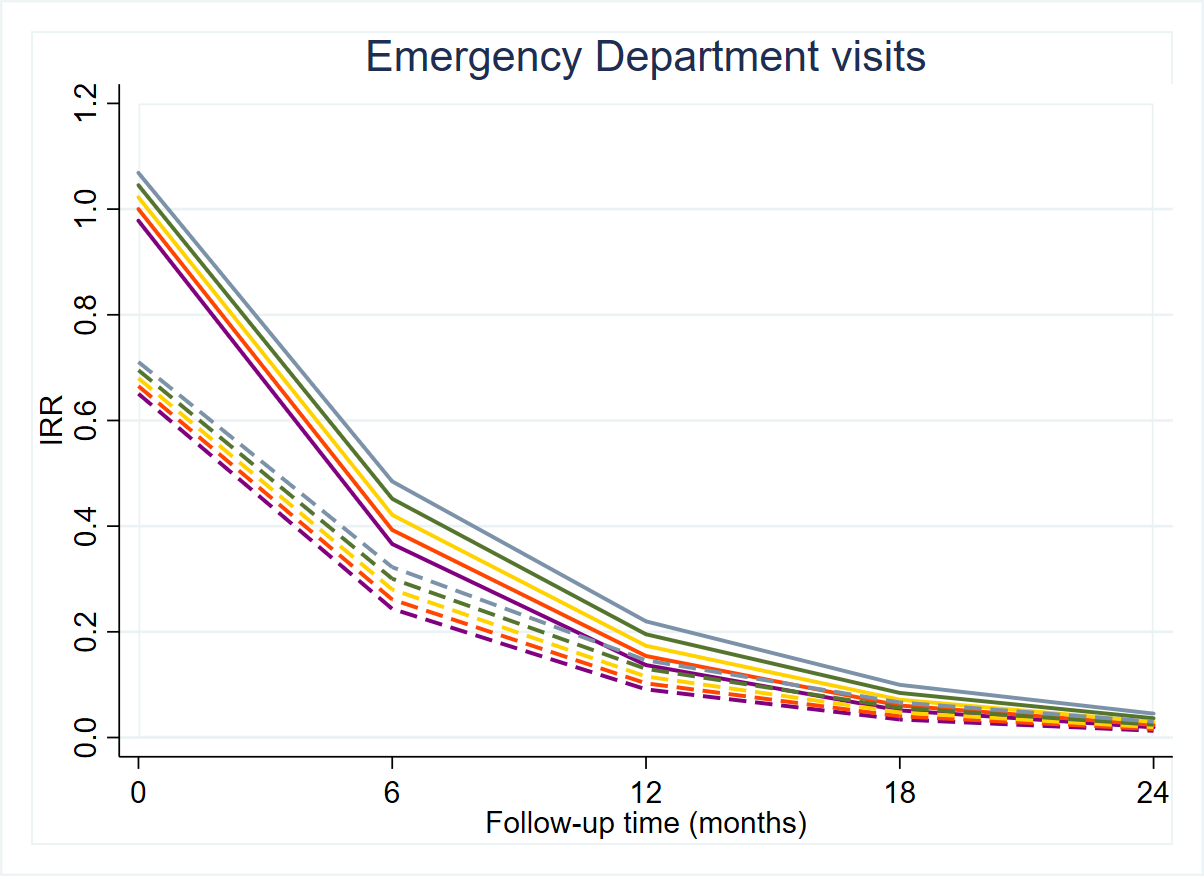

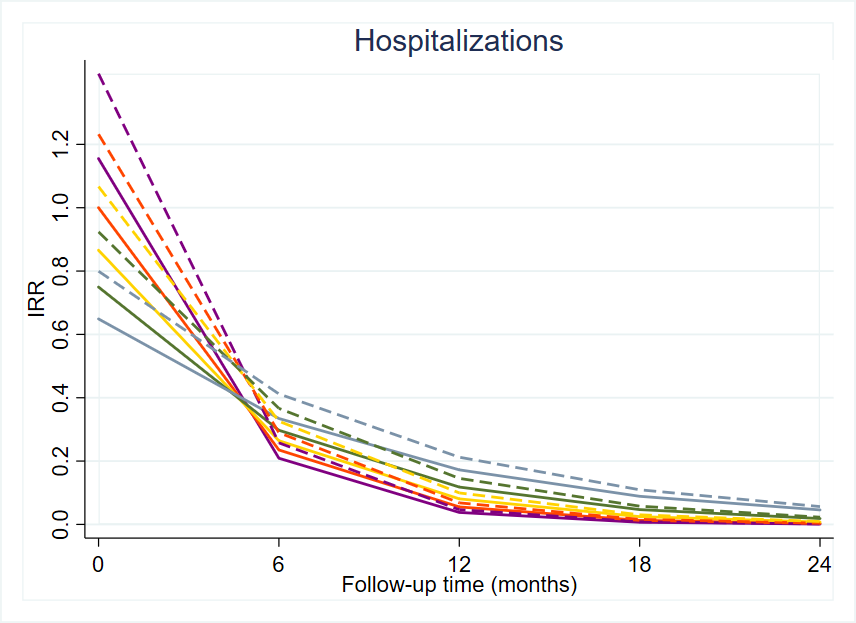

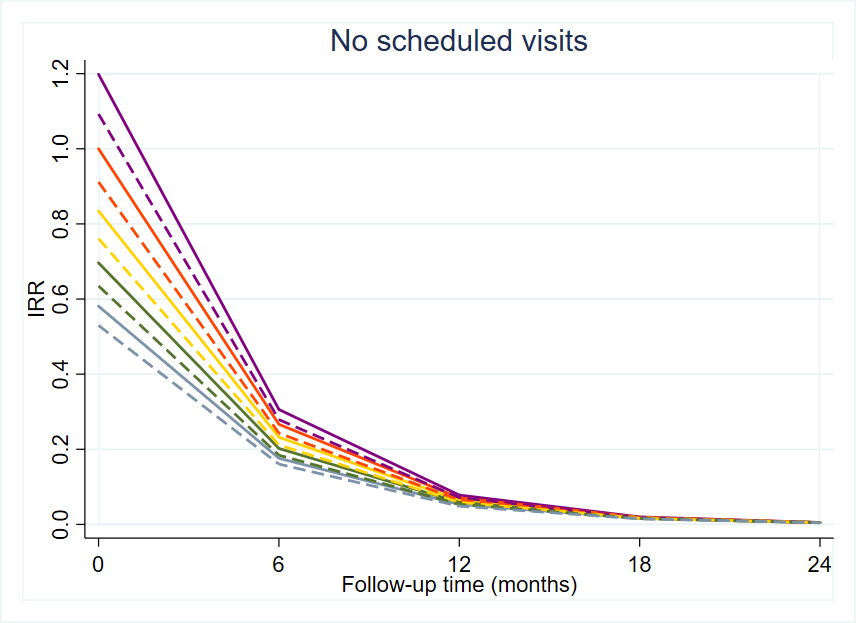
**

# **Online supplement 13. ZINB-estimated IRR for exacerbations (A), emergency department visits (B), hospitalizations (C), and unscheduled visits (D), stratified by BMI and treatment group.**

**A. Exacerbations requiring systemic corticosteroid**

|  | **Naïve** | | | | | **On biologics** | | | | |
| --- | --- | --- | --- | --- | --- | --- | --- | --- | --- | --- |
| **Time (months)** | **BMI** | | | | | **BMI** | | | | |
|  | **20** | **25** | **30** | **35** | **40** | **20** | **25** | **30** | **35** | **40** |
|  | **IRR  (95% CI)** | **IRR  (95% CI)** | **IRR  (95% CI)** | **IRR  (95% CI)** | **IRR  (95% CI)** | **IRR  (95% CI)** | **IRR  (95% CI)** | **IRR  (95% CI)** | **IRR  (95% CI)** | **IRR  (95% CI)** |
| 0 | 1  (0∙8; 1∙19) | Reference | 1  (0∙84; 1∙17) | 1∙01  (0∙77; 1∙25) | 1∙01  (0∙68; 1∙35) | 0∙66  (0∙49; 0∙82) | 0∙66  (0∙52; 0∙81) | 0∙67  (0∙5; 0∙83) | 0∙67  (0∙47; 0∙87) | 0∙67  (0∙42; 0∙92) |
| 6 | 0∙34  (0∙27; 0∙41) | 0∙36  (0∙3; 0∙41) | 0∙37  (0∙3; 0∙44) | 0∙39  (0∙28; 0∙49) | 0∙4  (0∙25; 0∙56) | 0∙23  (0∙17; 0∙28) | 0∙24  (0∙18; 0∙29) | 0∙25  (0∙19; 0∙3) | 0∙26  (0∙18; 0∙33) | 0∙27  (0∙16; 0∙37) |
| 12 | 0∙12  (0∙08; 0∙15) | 0∙13  (0∙1; 0∙15) | 0∙14  (0∙1; 0∙17) | 0∙15  (0∙09; 0∙21) | 0∙16  (0∙07; 0∙25) | 0∙08  (0∙05; 0∙1) | 0∙08  (0∙06; 0∙1) | 0∙09  (0∙07; 0∙11) | 0∙1  (0∙06; 0∙14) | 0∙11  (0∙05; 0∙16) |
| 18 | 0∙04  (0∙02; 0∙06) | 0∙04  (0∙03; 0∙06) | 0∙05  (0∙03; 0∙07) | 0∙06  (0∙03; 0∙09) | 0∙06  (0∙01; 0∙11) | 0∙03  (0∙02; 0∙04) | 0∙03  (0∙02; 0∙04) | 0∙03  (0∙02; 0∙04) | 0∙04  (0∙02; 0∙06) | 0∙04  (0∙01; 0∙07) |
| 24 | 0∙01  (0∙01; 0∙02) | 0∙02  (0∙01; 0∙02) | 0∙02  (0∙01; 0∙03) | 0∙02  (0∙01; 0∙04) | 0∙03  (0; 0∙05) | 0∙01  (0; 0∙01) | 0∙01  (0∙01; 0∙01) | 0∙01  (0∙01; 0∙02) | 0∙01  (0; 0∙02) | 0∙02  (0; 0∙03) |

*Multi-adjusted for baseline age, sex,* CRSwNP*, baseline eosinophils (n), baseline FEV1, baseline ACT, days on biologic therapy at baseline.*

**B. Emergency Department visits**

|  | **Naïve** | | | | | **On biologics** | | | | |
| --- | --- | --- | --- | --- | --- | --- | --- | --- | --- | --- |
| **Time (months)** | **BMI** | | | | | **BMI** | | | | |
|  | **20** | **25** | **30** | **35** | **40** | **20** | **25** | **30** | **35** | **40** |
|  | **IRR  (95% CI)** | **IRR  (95% CI)** | **IRR  (95% CI)** | **IRR  (95% CI)** | **IRR  (95% CI)** | **IRR  (95% CI)** | **IRR  (95% CI)** | **IRR  (95% CI)** | **IRR  (95% CI)** | **IRR  (95% CI)** |
| 0 | 0∙98  (0∙56; 1∙4) | Reference | 1∙02  (0∙69; 1∙35) | 1∙05  (0∙54; 1∙55) | 1∙07  (0∙33; 1∙81) | 0∙65  (0∙33; 0∙97) | 0∙67  (0∙38; 0∙95) | 0∙68  (0∙35; 1∙01) | 0∙7  (0∙25; 1∙14) | 0∙71  (0∙12; 1∙3) |
| 6 | 0∙37  (0∙22; 0∙51) | 0∙39  (0∙28; 0∙5) | 0∙42  (0∙29; 0∙55) | 0∙45  (0∙24; 0∙67) | 0∙48  (0∙16; 0∙81) | 0∙24  (0∙12; 0∙37) | 0∙26  (0∙15; 0∙38) | 0∙28  (0∙14; 0∙42) | 0∙3  (0∙12; 0∙49) | 0∙32  (0∙07; 0∙58) |
| 12 | 0∙14  (0∙08; 0∙19) | 0∙15  (0∙11; 0∙2) | 0∙17  (0∙11; 0∙23) | 0∙2  (0∙09; 0∙3) | 0∙22  (0∙06; 0∙38) | 0∙09  (0∙04; 0∙14) | 0∙1  (0∙05; 0∙15) | 0∙12  (0∙06; 0∙17) | 0∙13  (0∙05; 0∙21) | 0∙15  (0∙03; 0∙27) |
| 18 | 0∙05  (0∙03; 0∙07) | 0∙06  (0∙04; 0∙08) | 0∙07  (0∙04; 0∙1) | 0∙08  (0∙03; 0∙14) | 0∙1  (0∙01; 0∙19) | 0∙03  (0∙01; 0∙06) | 0∙04  (0∙02; 0∙06) | 0∙05  (0∙02; 0∙07) | 0∙06  (0∙02; 0∙1) | 0∙07  (0; 0∙13) |
| 24 | 0∙02  (0∙01; 0∙03) | 0∙02  (0∙02; 0∙03) | 0∙03  (0∙02; 0∙04) | 0∙04  (0∙01; 0∙06) | 0∙05  (0; 0∙09) | 0∙01  (0; 0∙02) | 0∙02  (0∙01; 0∙02) | 0∙02  (0∙01; 0∙03) | 0∙02  (0; 0∙04) | 0∙03  (0; 0∙06) |

*Multi-adjusted for baseline age, sex,* CRSwNP*, baseline eosinophils (n), baseline FEV1, baseline ACT, days on biologic therapy at baseline.*

**C. Hospitalizations**

|  | **Naïve** | | | | | **On biologics** | | | | |
| --- | --- | --- | --- | --- | --- | --- | --- | --- | --- | --- |
| **Time (months)** | **BMI** | | | | | **BMI** | | | | |
|  | **20** | **25** | **30** | **35** | **40** | **20** | **25** | **30** | **35** | **40** |
|  | **IRR  (95% CI)** | **IRR  (95% CI)** | **IRR  (95% CI)** | **IRR  (95% CI)** | **IRR  (95% CI)** | **IRR  (95% CI)** | **IRR  (95% CI)** | **IRR  (95% CI)** | **IRR  (95% CI)** | **IRR  (95% CI)** |
| 0 | 1∙16  (0∙62; 1∙69) | Reference | 0∙87  (0∙55; 1∙18) | 0∙75  (0∙41; 1∙09) | 0∙65  (0∙26; 1∙04) | 1∙42  (0∙8; 2∙05) | 1∙23  (0∙72; 1∙74) | 1∙07  (0∙56; 1∙58) | 0∙92  (0∙37; 1∙48) | 0∙8  (0∙19; 1∙41) |
| 6 | 0∙21  (0∙04; 0∙38) | 0∙24  (0∙12; 0∙35) | 0∙26  (0∙14; 0∙38) | 0∙3  (0∙07; 0∙52) | 0∙33  (0; 0∙72) | 0∙26  (0; 0∙53) | 0∙29  (0∙08; 0∙5) | 0∙33  (0∙14; 0∙51) | 0∙37  (0∙11; 0∙62) | 0∙41  (0; 0∙84) |
| 12 | 0∙04  (0; 0∙1) | 0∙06  (0; 0∙11) | 0∙08  (0∙02; 0∙14) | 0∙12  (0; 0∙26) | 0∙17  (0; 0∙51) | 0∙05  (0; 0∙14) | 0∙07  (0; 0∙15) | 0∙1  (0∙01; 0∙18) | 0∙15  (0; 0∙31) | 0∙21  (0; 0∙59) |
| 18 | 0∙01  (0; 0∙02) | 0∙01  (0; 0∙03) | 0∙02  (0; 0∙05) | 0∙05  (0; 0∙13) | 0∙09  (0; 0∙34) | 0∙01  (0; 0∙03) | 0∙02  (0; 0∙05) | 0∙03  (0; 0∙07) | 0∙06  (0; 0∙15) | 0∙11  (0; 0∙39) |
| 24 | 0  (0; 0∙01) | 0  (0; 0∙01) | 0∙01  (0; 0∙02) | 0∙02  (0; 0∙06) | 0∙05  (0; 0∙21) | 0  (0; 0∙01) | 0  (0; 0∙01) | 0∙01  (0; 0∙02) | 0∙02  (0; 0∙07) | 0∙06  (0; 0∙25) |

*Multi-adjusted for baseline age, sex,* CRSwNP*, baseline eosinophils (n), baseline FEV1, baseline ACT, days on biologic therapy at baseline.*

**D. Unscheduled visits**

|  | **Naïve** | | | | | **On biologics** | | | | |
| --- | --- | --- | --- | --- | --- | --- | --- | --- | --- | --- |
| **Time (months)** | **BMI** | | | | | **BMI** | | | | |
|  | **20** | **25** | **30** | **35** | **40** | **20** | **25** | **30** | **35** | **40** |
|  | **IRR  (95% CI)** | **IRR  (95% CI)** | **IRR  (95% CI)** | **IRR  (95% CI)** | **IRR  (95% CI)** | **IRR  (95% CI)** | **IRR  (95% CI)** | **IRR  (95% CI)** | **IRR  (95% CI)** | **IRR  (95% CI)** |
| 0 | 1∙2  (0∙9; 1∙5) | Reference | 0∙83  (0∙63; 1∙04) | 0∙7  (0∙46; 0∙93) | 0∙58  (0∙32; 0∙84) | 1∙09  (0∙7; 1∙48) | 0∙91  (0∙6; 1∙22) | 0∙76  (0∙47; 1∙05) | 0∙63  (0∙35; 0∙92) | 0∙53  (0∙24; 0∙82) |
| 6 | 0∙31  (0∙21; 0∙41) | 0∙27  (0∙2; 0∙33) | 0∙23  (0∙17; 0∙29) | 0∙2  (0∙13; 0∙28) | 0∙18  (0∙09; 0∙26) | 0∙28  (0∙16; 0∙4) | 0∙24  (0∙15; 0∙34) | 0∙21  (0∙13; 0∙3) | 0∙18  (0∙1; 0∙27) | 0∙16  (0∙07; 0∙25) |
| 12 | 0∙08  (0∙03; 0∙12) | 0∙07  (0∙04; 0∙1) | 0∙06  (0∙04; 0∙09) | 0∙06  (0∙03; 0∙09) | 0∙05  (0∙01; 0∙09) | 0∙07  (0∙03; 0∙12) | 0∙06  (0∙03; 0∙1) | 0∙06  (0∙03; 0∙09) | 0∙05  (0∙02; 0∙09) | 0∙05  (0∙01; 0∙09) |
| 18 | 0∙02  (0; 0∙04) | 0∙02  (0∙01; 0∙03) | 0∙02  (0∙01; 0∙03) | 0∙02  (0; 0∙03) | 0∙02  (0; 0∙03) | 0∙02  (0; 0∙03) | 0∙02  (0∙01; 0∙03) | 0∙02  (0∙01; 0∙03) | 0∙02  (0; 0∙03) | 0∙01  (0; 0∙03) |
| 24 | 0∙01  (0; 0∙01) | 0∙01  (0; 0∙01) | 0  (0; 0∙01) | 0  (0; 0∙01) | 0  (0; 0∙01) | 0  (0; 0∙01) | 0  (0; 0∙01) | 0  (0; 0∙01) | 0  (0; 0∙01) | 0  (0; 0∙01) |

*Multi-adjusted for baseline age, sex,* CRSwNP*, baseline eosinophils (n), baseline FEV1, baseline ACT, days on biologic therapy at baseline.*

**Online supplement 14: Predicted rate of ACT, AQLQ and FEV_1_% improvement over time by treatment group and BMI category.**

Predicted trajectories of ACT score, AQLQ score, and FEV1% predicted according to BMI, modelled as a continuous variable. Separate panels show patients who were biologic-naïve at baseline (left) and those already on biologics (right). Post-estimation margins were used to derive values at representative BMI levels, which are shown as separate curves with 95% confidence intervals.


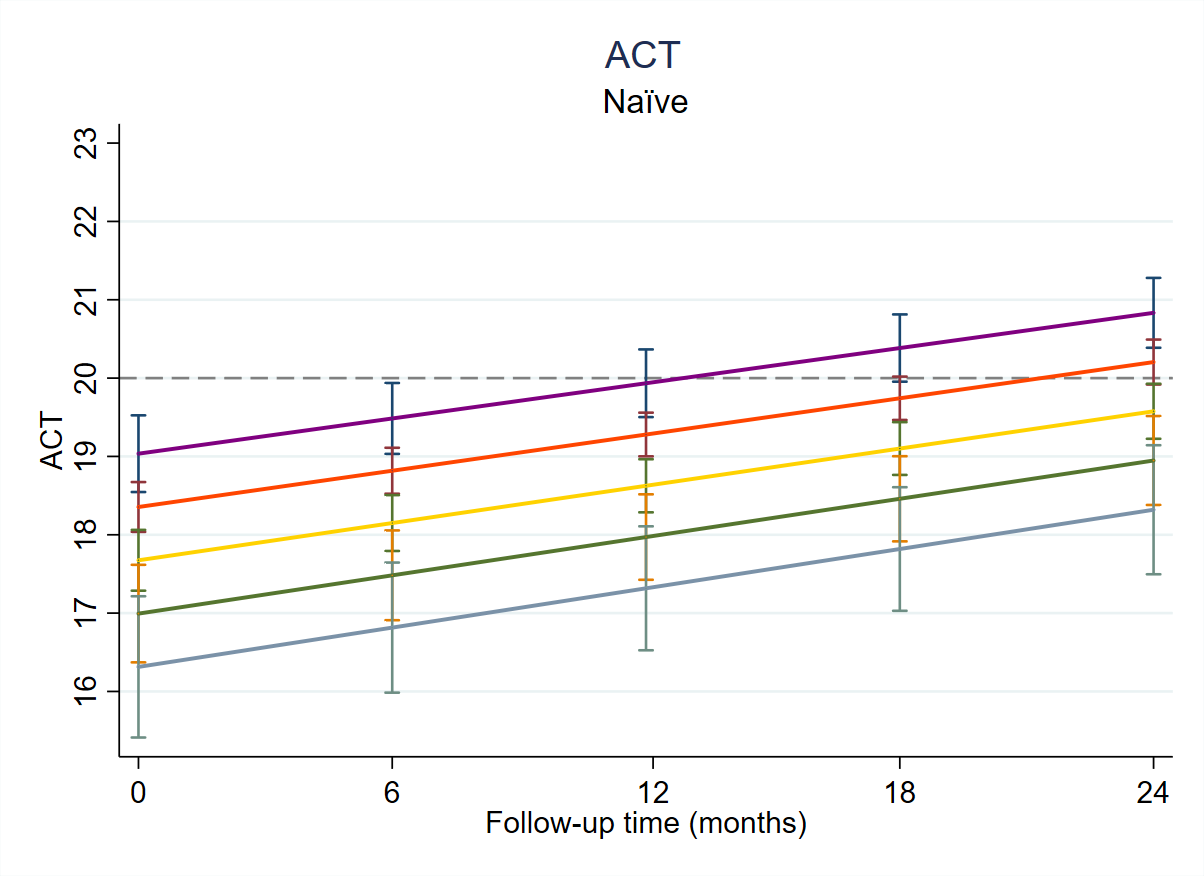

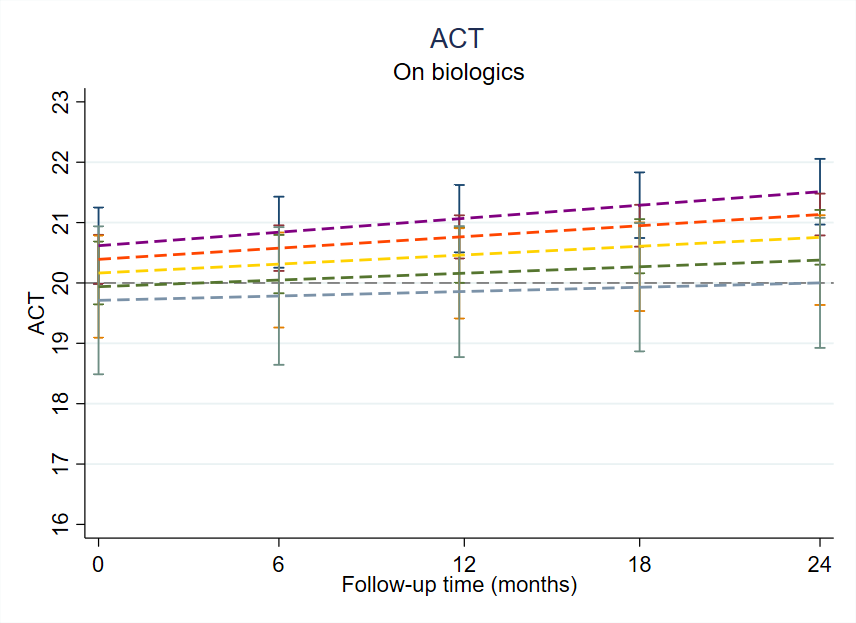

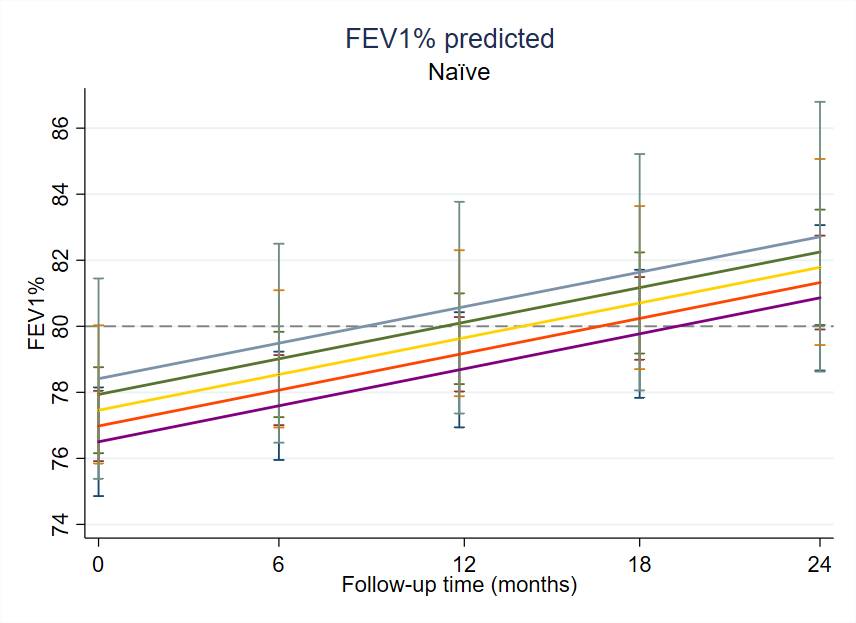

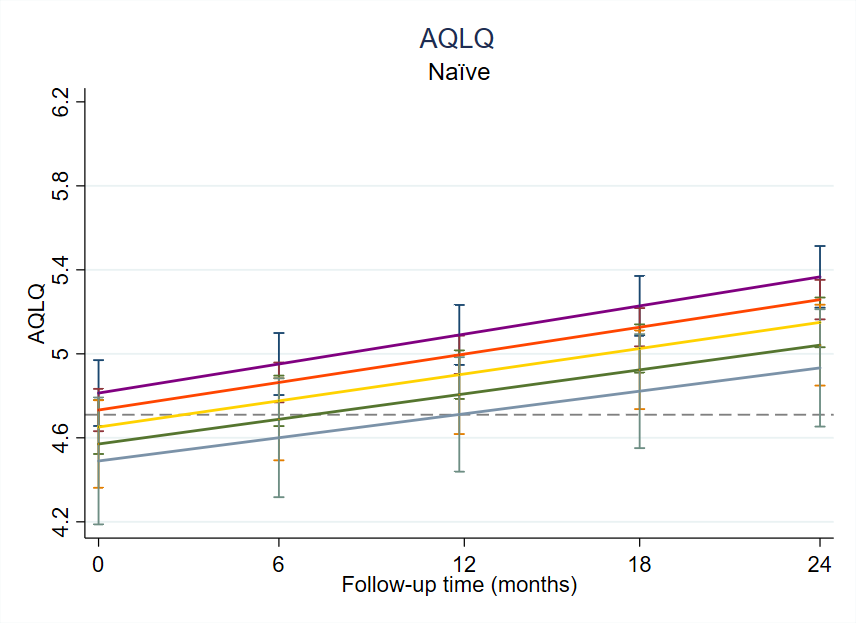

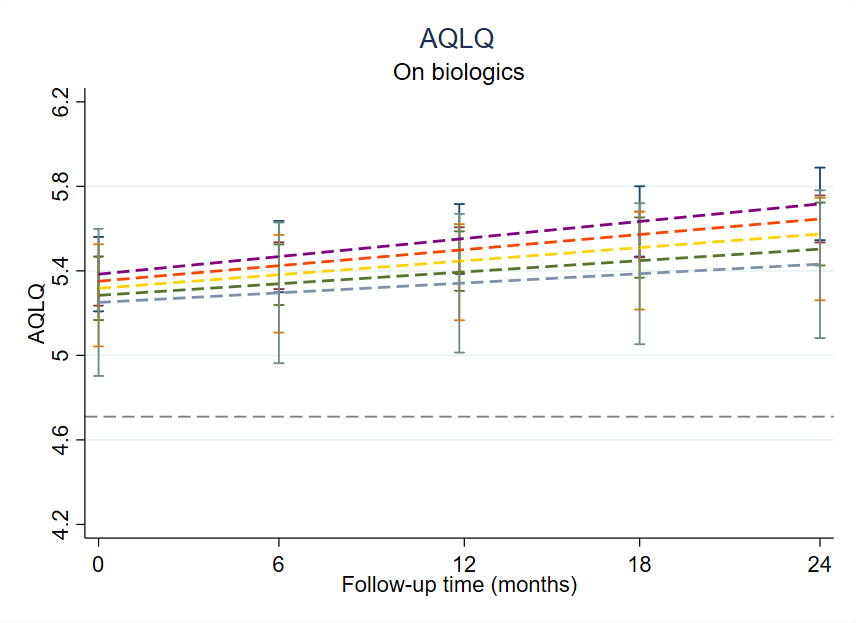

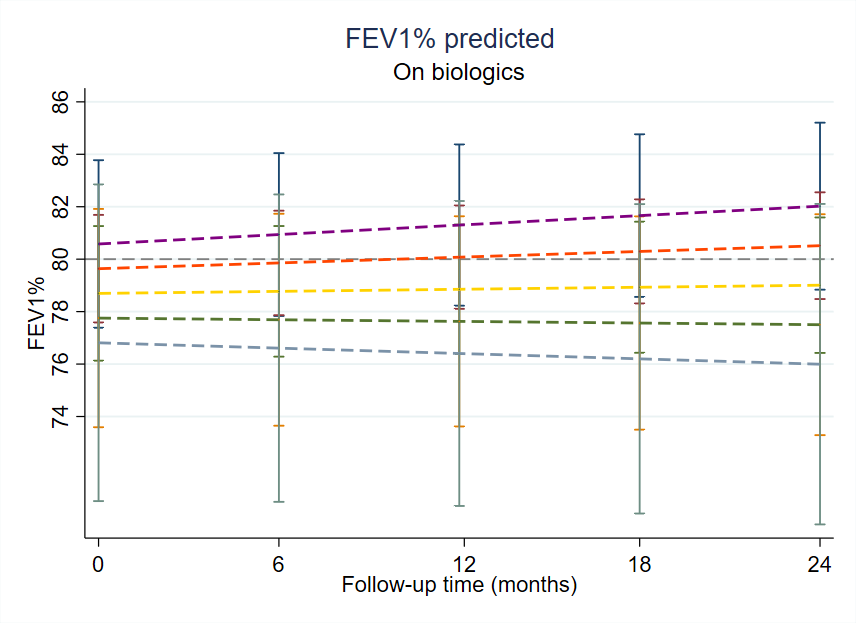


**
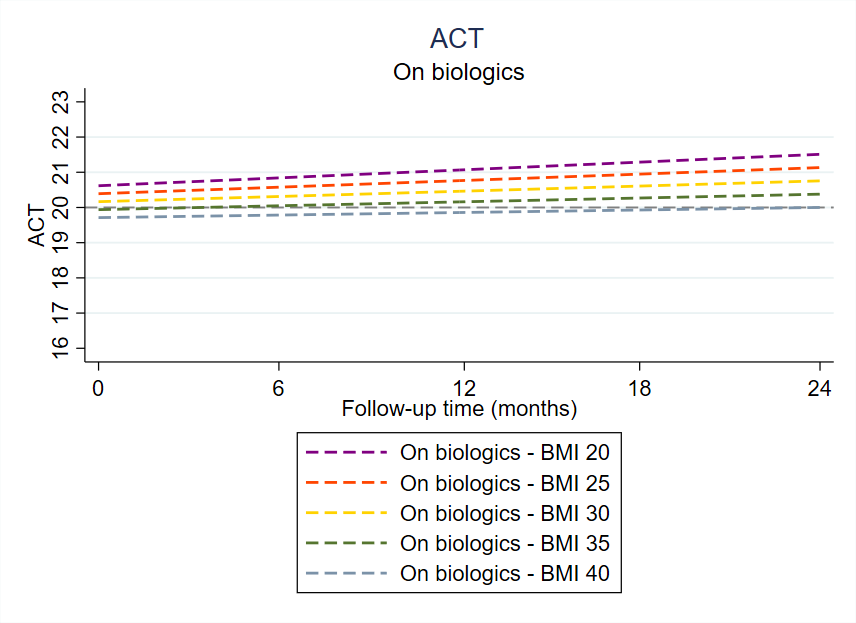
**


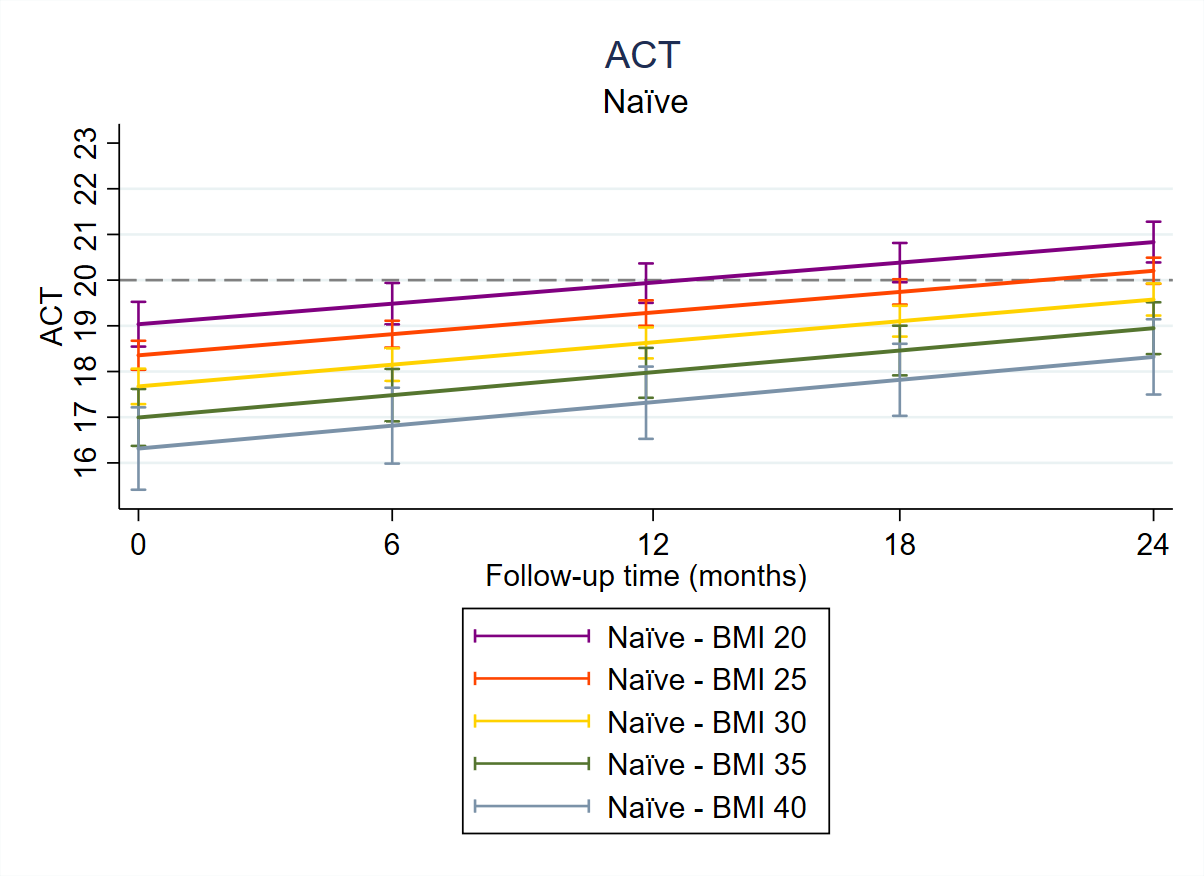


**Online supplement 15. Linear mixed-effects models-estimated β changes for ACT (A), FEV1 % (B), AQLQ (C), stratified by BMI and treatment group.**

**A. ACT**

|  | **Naïve** | | | | | **On biologics** | | | | |
| --- | --- | --- | --- | --- | --- | --- | --- | --- | --- | --- |
| **Time (months)** | **BMI** | | | | | **BMI** | | | | |
|  | **20** | **25** | **30** | **35** | **40** | **20** | **25** | **30** | **35** | **40** |
|  | **β (95% CI)** | **β (95% CI)** | **β (95% CI)** | **β (95% CI)** | **β (95% CI)** | **β (95% CI)** | **β (95% CI)** | **β (95% CI)** | **β (95% CI)** | **β (95% CI)** |
| 0 | 0∙68  (0∙37; 0∙99) | Reference | -0∙68  (-0∙99; -0∙37) | -1∙36  (-1∙97; -0∙75) | -2∙04  (-2∙96; -1∙12) | 0∙23  (-0∙19; 0∙64) | Reference | -0∙23  (-0∙64; 0∙19) | -0∙45  (-1∙28; 0∙37) | -0∙68  (-1∙92; 0∙56) |
| 6 | 1∙13  (0∙84; 1∙42) | 0∙46  (0∙40; 0∙52) | -0∙2  (-0∙50; 0∙09) | -0∙87  (-1∙44; -0∙3) | -1∙54  (-2∙39; -0∙69) | 0∙45  (0∙07; 0∙83) | 0∙19  (0∙13; 0∙25) | -0∙08  (-0∙47; 0∙31) | -0∙34  (-1∙12; 0∙43) | -0∙61  (-1∙76; 0∙55) |
| 12 | 1∙58  (1∙29; 1∙87) | 0∙93  (0∙81; 1∙04) | 0∙27  (-0∙03; 0∙57) | -0∙38  (-0∙94; 0∙17) | -1∙04  (-1∙86; -0∙22) | 0∙67  (0∙30; 1∙05) | 0∙37  (0∙25; 0∙49) | 0∙07  (-0∙32; 0∙46) | -0∙23  (-0∙98; 0∙51) | -0∙53  (-1∙64; 0∙57) |
| 18 | 2∙03  (1∙70; 2∙35) | 1∙39  (1∙21; 1∙56) | 0∙75  (0∙43; 1∙07) | 0∙1  (-0∙46; 0∙67) | -0∙54  (-1∙36; 0∙29) | 0∙9  (0∙50; 1∙29) | 0∙56  (0∙38; 0∙74) | 0∙22  (-0∙19; 0∙62) | -0∙12  (-0∙87; 0∙62) | -0∙46  (-1∙56; 0∙63) |
| 24 | 2∙48  (2∙10; 2∙85) | 1∙85  (1∙61; 2∙09) | 1∙22  (0∙86; 1∙58) | 0∙59  (-0∙00; 1∙19) | -0∙04  (-0∙90; 0∙83) | 1∙12  (0∙69; 1∙55) | 0∙74  (0∙50; 0∙98) | 0∙37  (-0∙07; 0∙8) | -0∙01  (-0∙78; 0∙75) | -0∙39  (-1∙50; 0∙72) |

*Multi-adjusted for baseline age, sex,* CRSwNP*, baseline eosinophils (n), baseline FEV1, , baseline number of exacerbations (n), days on biologic therapy at baseline.*

**B. FEV1%**

|  | **Naïve** | | | | | **On biologics** | | | | |
| --- | --- | --- | --- | --- | --- | --- | --- | --- | --- | --- |
| **Time (months)** | **BMI** | | | | | **BMI** | | | | |
|  | **20** | **25** | **30** | **35** | **40** | **20** | **25** | **30** | **35** | **40** |
|  | **β (95% CI)** | **β (95% CI)** | **β (95% CI)** | **β (95% CI)** | **β (95% CI)** | **β (95% CI)** | **β (95% CI)** | **β (95% CI)** | **β (95% CI)** | **β (95% CI)** |
| 0 | -0∙48  (-1∙51; 0∙55) | Reference | 0∙48  (-0∙55; 1∙51) | 0∙96  (-1∙11; 3∙02) | 1∙43  (-1∙66; 4∙53) | 0∙94  (-1∙10; 2∙99) | Reference | -0∙94  (-2∙99; 1∙1) | -1∙88  (-5∙98; 2∙21) | -2∙83  (-8∙96; 3∙31) |
| 6 | 0∙61  (-0∙46; 1∙68) | 1∙09  (0∙81; 1∙36) | 1∙56  (0∙51; 2∙61) | 2∙03  (-0∙03; 4∙1) | 2∙51  (-0∙57; 5∙59) | 1∙3  (-0∙70; 3∙3) | 0∙22  (-0∙05; 0∙49) | -0∙86  (-2∙88; 1∙15) | -1∙95  (-5∙94; 2∙05) | -3∙03  (-9∙01; 2∙95) |
| 12 | 1∙7  (0∙44; 2∙97) | 2∙17  (1∙62; 2∙73) | 2∙64  (1∙46; 3∙83) | 3∙11  (0∙90; 5∙32) | 3∙58  (0∙30; 6∙86) | 1∙66  (-0∙38; 3∙7) | 0∙44  (-0∙10; 0∙98) | -0∙79  (-2∙84; 1∙26) | -2∙01  (-5∙99; 1∙98) | -3∙23  (-9∙18; 2∙71) |
| 18 | 2∙79  (1∙24; 4∙35) | 3∙26  (2∙43; 4∙09) | 3∙72  (2∙33; 5∙11) | 4∙19  (1∙71; 6∙67) | 4∙66  (1∙01; 8∙31) | 2∙02  (-0∙14; 4∙19) | 0∙66  (-0∙16; 1∙47) | -0∙71  (-2∙86; 1∙44) | -2∙07  (-6∙14; 2) | -3∙44  (-9∙48; 2∙6) |
| 24 | 3∙88  (1∙98; 5∙78) | 4∙34  (3∙24; 5∙45) | 4∙81  (3∙16; 6∙45) | 5∙27  (2∙43; 8∙11) | 5∙73  (1∙58; 9∙89) | 2∙38  (0∙02; 4∙75) | 0∙88  (-0∙21; 1∙96) | -0∙63  (-2∙93; 1∙67) | -2∙14  (-6∙37; 2∙1) | -3∙64  (-9∙90; 2∙62) |

*Multi-adjusted for baseline age, sex,* CRSwNP*, baseline eosinophils (n), baseline FEV1, , baseline number of exacerbations (n), days on biologic therapy at baseline.*

**C. AQLQ**

|  | **Naïve** | | | | | **On biologics** | | | | |
| --- | --- | --- | --- | --- | --- | --- | --- | --- | --- | --- |
| **Time (months)** | **BMI** | | | | | **BMI** | | | | |
|  | **20** | **25** | **30** | **35** | **40** | **20** | **25** | **30** | **35** | **40** |
|  | **β (95% CI)** | **β (95% CI)** | **β (95% CI)** | **β (95% CI)** | **β (95% CI)** | **β (95% CI)** | **β (95% CI)** | **β (95% CI)** | **β (95% CI)** | **β (95% CI)** |
| 0 | 0∙08  (-0∙02; 0∙18) | Reference | -0∙08  (-0∙18; 0∙02) | -0∙16  (-0∙37; 0∙04) | -0∙24  (-0∙55; 0∙06) | 0∙03  (-0∙08; 0∙15) | Reference | -0∙03  (-0∙15; 0∙08) | -0∙07  (-0∙30; 0∙16) | -0∙1  (-0∙45; 0∙25) |
| 6 | 0∙22  (0∙12; 0∙32) | 0∙13  (0∙11; 0∙15) | 0∙04  (-0∙05; 0∙14) | -0∙04  (-0∙24; 0∙15) | -0∙13  (-0∙42; 0∙16) | 0∙12  (0∙01; 0∙23) | 0∙07  (0∙06; 0∙09) | 0∙03  (-0∙08; 0∙14) | -0∙01  (-0∙23; 0∙21) | -0∙05  (-0∙39; 0∙28) |
| 12 | 0∙36  (0∙26; 0∙46) | 0∙26  (0∙23; 0∙30) | 0∙17  (0∙07; 0∙27) | 0∙07  (-0∙11; 0∙26) | -0∙02  (-0∙30; 0∙26) | 0∙2  (0∙09; 0∙31) | 0∙15  (0∙11; 0∙18) | 0∙1  (-0∙02; 0∙21) | 0∙04  (-0∙18; 0∙26) | -0∙01  (-0∙34; 0∙32) |
| 18 | 0∙5  (0∙39; 0∙60) | 0∙39  (0∙34; 0∙45) | 0∙29  (0∙19; 0∙40) | 0∙19  (0∙00; 0∙38) | 0∙09  (-0∙19; 0∙37) | 0∙28  (0∙16; 0∙41) | 0∙22  (0∙17; 0∙27) | 0∙16  (0∙04; 0∙28) | 0∙1  (-0∙13; 0∙33) | 0∙04  (-0∙30; 0∙37) |
| 24 | 0∙63  (0∙52; 0∙75) | 0∙53  (0∙46; 0∙59) | 0∙42  (0∙30; 0∙53) | 0∙31  (0∙11; 0∙51) | 0∙2  (-0∙09; 0∙49) | 0∙37  (0∙23; 0∙50) | 0∙29  (0∙22; 0∙36) | 0∙22  (0∙09; 0∙36) | 0∙15  (-0∙09; 0∙39) | 0∙08  (-0∙27; 0∙44) |

*Multi-adjusted for baseline age, sex,* CRSwNP*, baseline eosinophils (n), baseline FEV1, , baseline number of exacerbations (n), days on biologic therapy at baseline.*

**Online supplement 16. Baseline Clinical Remission Status According to BMI and Biologic Exposure**

Remission status is defined according to SANI criteria (online supplement 2).

|  | **Biologic-naïve at inclusion** | | | | **On biologics at inclusion** | | | |
| --- | --- | --- | --- | --- | --- | --- | --- | --- |
|  | **Total** | **BMI<30** | **BMI≥30** | **P** | **Total** | **BMI<30** | **BMI≥30** | ***P*** |
|  | N=711 | N=565 | N=146 |  | N=599 | N=494 | N=105 |  |
| **Clinical remission** |  |  |  | 0·612 |  |  |  | 0·203 |
| No remission | 566 | 450 (79·65 %) | 116 (79·45%) |  | 319 | 255 (51·62%) | 64 (60·95%) |  |
| Partial remission | 122 | 95 (16·81%) | 27 (18·49%) |  | 176 | 149 (30·16%) | 27 (25·71%) |  |
| Complete remission | 23 | 20 (3·54%) | 3 (2·05%) |  | 104 | 90 (18·22%) | 14 (13·33%) |  |

Baseline clinical remission status, defined according to SANI criteria. Frequencies of no remission, partial remission, and complete remission are reported for biologic-naïve and on-biologic patients, stratified by BMI category.

**Online supplement 17. Dropouts and related adverse events.**

|  | **Biologic-naïve at inclusion** | | | | | **On biologics at inclusion** | | | |
| --- | --- | --- | --- | --- | --- | --- | --- | --- | --- |
|  | | **Total** | **BMI<30** | **BMI≥30** | ***P*** | **Total** | **BMI<30** | **BMI≥30** | ***P*** |
|  | | N=1·118 | N=893 | N=225 |  | N=980 | N=800 | N=180 |  |
| **Patients with baseline data only** | | 233 (20·84%) | 187 (20·94%) | 46 (20·44%) | 0·870 | 212 (21·63%) | 176 (22·00%) | 36 (20·00%) | 0·556 |
| *AE* | | 2 (22·22%) | 2 (28·57%) | 0 (0%) | 0·391 | 1 (100%) | 1 (100%) | 0 | N/A |
| **Patients lost within 12 months** | | 262 (29·60%) | 203 (28·75%) | 59 (32·96%) | 0·271 | 226 (29·43%) | 184 (29·49%) | 42 (29·17%) | 0·939 |
| *AE* | | 2 (28·57%) | 1 (20·00%) | 1 (50·00%) | 0·427 | 0 | 0 | 0 | N/A |

Number and proportion of patients lost to follow-up, stratified by treatment status and BMI category. The table reports patients observed only at baseline, those lost within 12 months, and cases in which dropout was associated with adverse events (AE). Data are shown as numbers and percentages.

**Online supplement 18. Adverse events.**

|  | | **Biologic-naïve at inclusion** | | | | **On biologics at inclusion** | | | |
| --- | --- | --- | --- | --- | --- | --- | --- | --- | --- |
|  | **Total** | | **BMI<30** | **BMI≥30** | ***P*** | **Total** | **BMI<30** | **BMI≥30** | ***P*** |
|  | N=1·118 | | N=893 | N=225 |  | N=980 | N=800 | N=180 |  |
| **Total** | 45 | | 40 | 5 | ***0·135*** | 29 | 19 | 10 | ***0·411*** |
| **Death** | 1 | | 0 (0%) | 1 (20·00%) |  | 0 | 0 (0%) | 0 (0%) |  |
| **Fever** | 1 | | 1 (2·50%) | 0 (0%) |  | 1 | 0 (0%) | 1 (10%) |  |
| **Headache** | 2 | | 2 (3·23%) | 0 (0%) |  | 3 | 1 (5·26%) | 2 (20·00%) |  |
| **Dermatological** | 8 | | 7 (17·50%) | 1 (20·00%) |  | 2 | 1 (5·26%) | 1 (10·00%) |  |
| **Joint disease** | 13 | | 13 (32·50%) | 0 (0%) |  | 3 | 3 (15·79%) | 0 (0%) |  |
| **EGPA** | 1 | | 1 (2·50%) | 0 (0%) |  | 2 | 2 (10·53%) | 0 (0%) |  |
| **Respiratory symptoms** | 1 | | 1 (2·50%) | 0 (0%) |  | 0 | 0 (0%) | 0 (0%) |  |
| **Peripheral blood hypereosinophilia** | 3 | | 3 (4·84%) | 0 (0%) |  | 7 | 5 (26·32%) | 2 (20·00%) |  |
| **Mixed** | 5 | | 5 (8·06%) | 0 (0%) |  | 3 | 1 (5·26%) | 2 (20·00%) |  |
| **Miscellaneous** | 7 | | 5 (12·50%) | 2 (40·00%) |  | 7 | 5 (26·32%) | 2 (20·00%) |  |
| **Unspecified** | 3 | | 2 (5·00%) | 1 (20·00%) |  | 1 | 1 (5·26%) | 0 (0%) |  |

Distribution of adverse events reported during follow-up, stratified by treatment status (biologic-naïve vs. on biologics) and BMI category (<30 vs. ≥30). Data are presented as absolute numbers for each event category (death, fever, headache, dermatological, joint disease, EGPA, respiratory symptoms, hypereosinophilia, mixed, miscellaneous, unspecified). EGPA, Eosinophilic granulomatosis with polyangiitis. Data reported as numbers (%).

**Online supplement 19. Follow-up time according to BMI and Biologic Exposure.**

|  | | **Biologic-naïve at inclusion** | | | | **On biologics at inclusion** | | | |
| --- | --- | --- | --- | --- | --- | --- | --- | --- | --- |
|  | **Total** | | **BMI<30** | **BMI≥30** | ***P*** | **Total** | **BMI<30** | **BMI≥30** | ***P*** |
|  | N=1·118 | | N=893 | N=225 |  | N=980 | N=800 | N=180 |  |
| **Time (days)** | 671  (329 - 1339) | | 671·5  (339 - 1339) | 651  (245 - 1340) | 0·151 | 757  (343 - 1439) | 761  (336 - 1464) | 718  (357 - 1298) | 0·439 |

Time (days) is reported as median (IQR).

Follow-up duration reported as median and interquartile range (IQR), stratified by treatment status (biologic-naïve vs. on biologics) and BMI category (<30 vs. ≥30). P values indicate comparisons between BMI categories.

**Online supplement 20. Cox proportional hazards models estimating hazard ratios for incident partial or complete remission, stratified by treatment group and accounting for random effects due to the multicentre study design.**

|  | **Naive** | | **On biologics** | |
| --- | --- | --- | --- | --- |
|  | **HR (95% CI)** | ***P*** | **HR (95% CI)** | ***P*** |
| **BMI≥30** | 0∙83 (0∙52, 1∙32) | *0∙437* | 1∙34 (0∙61, 2∙94) | *0∙456* |

**Online supplement 21. Cox proportional hazards models estimating hazard ratios for incident partial or complete remission, excluding biologic-naïve patients who initiated treatment within one month prior to enrolment (n=71).**

|  | **Naive** | |
| --- | --- | --- |
|  | **HR (95% CI)** | ***P*** |
| **BMI≥30** | 0∙89 (0∙57, 1∙38) | *0∙599* |

**Online supplement 22. Cox model–estimated hazard ratios for incident partial or complete remission, stratified by treatment group, with the risk period starting at 12 months.**

|  | **Naive** | | **On biologics** | |
| --- | --- | --- | --- | --- |
|  | **HR (95% CI)** | ***P*** | **HR (95% CI)** | ***P*** |
| **BMI≥30** | 0∙90 (0∙58, 1∙39) | *0∙630* | 0∙94 (0∙45, 1∙94) | *0∙867* |
| **Age** | 1∙00 (0∙98, 1∙01) | *0∙572* | 1∙00 (0∙97, 1∙02) | *0∙906* |
| **Sex (Female)** | 0∙97 (0∙69, 1∙37) | *0∙860* | 0∙67 (0∙40, 1∙14) | *0∙145* |
| **CRwNP** | 0∙96 (0∙69, 1∙33) | *0∙801* | 1∙18 (0∙70, 1∙98) | *0∙542* |
| **BEC** | 1∙02 (0∙99, 1∙05) | *0∙108* | 0∙74 (0∙37, 1∙46) | *0∙380* |
| **FEV_1_% (pre-bronchodilator)** | 1∙00 (1∙00, 1∙01) | ***0∙043*** | 1∙00 (0∙99, 1∙01) | *0∙515* |
| **ACT** | 0∙99 (0∙96, 1∙03) | *0∙690* | 1∙04 (0∙98, 1∙10) | *0∙173* |
| **Exacerbations** | 0∙99 (0∙92, 1∙05) | *0∙667* | 1∙01 (0∙87, 1∙18) | *0∙895* |
| **Days on biologic therapy** | **-** | *-* | 1∙00 (0∙99, 1∙00) | *0∙119* |

BMI, body mass index; CRwNP, chronic rhinosinusitis with nasal polyps; BEC, blood eosinophil count; FEV_1_%, forced expiratory volume at 1 second.

|  | **Naive** | | **On biologics** | |
| --- | --- | --- | --- | --- |
|  | **HR (95% CI)** | ***P*** | **HR (95% CI)** | ***P*** |
| **BMI** | 0∙99 (0∙96, 1∙02) | *0∙483* | 0∙95 (0∙90, 1∙01) | *0∙132* |

BMI, body mass index; CRwNP, chronic rhinosinusitis with nasal polyps; BEC, blood eosinophil count; FEV_1_%, forced expiratory volume at 1 second.

**Online supplement 23. Restricted cubic spline–adjusted Cox proportional hazards model for the association between BMI and complete or partial remission.**

The figure shows the association between BMI and the hazard of achieving complete or partial remission in biologic-naïve patients. The red solid line represents the spline-based hazard ratio, and the shaded area indicates the 95% confidence interval. The blue dashed line represents the hazard ratio from a linear BMI term.

**
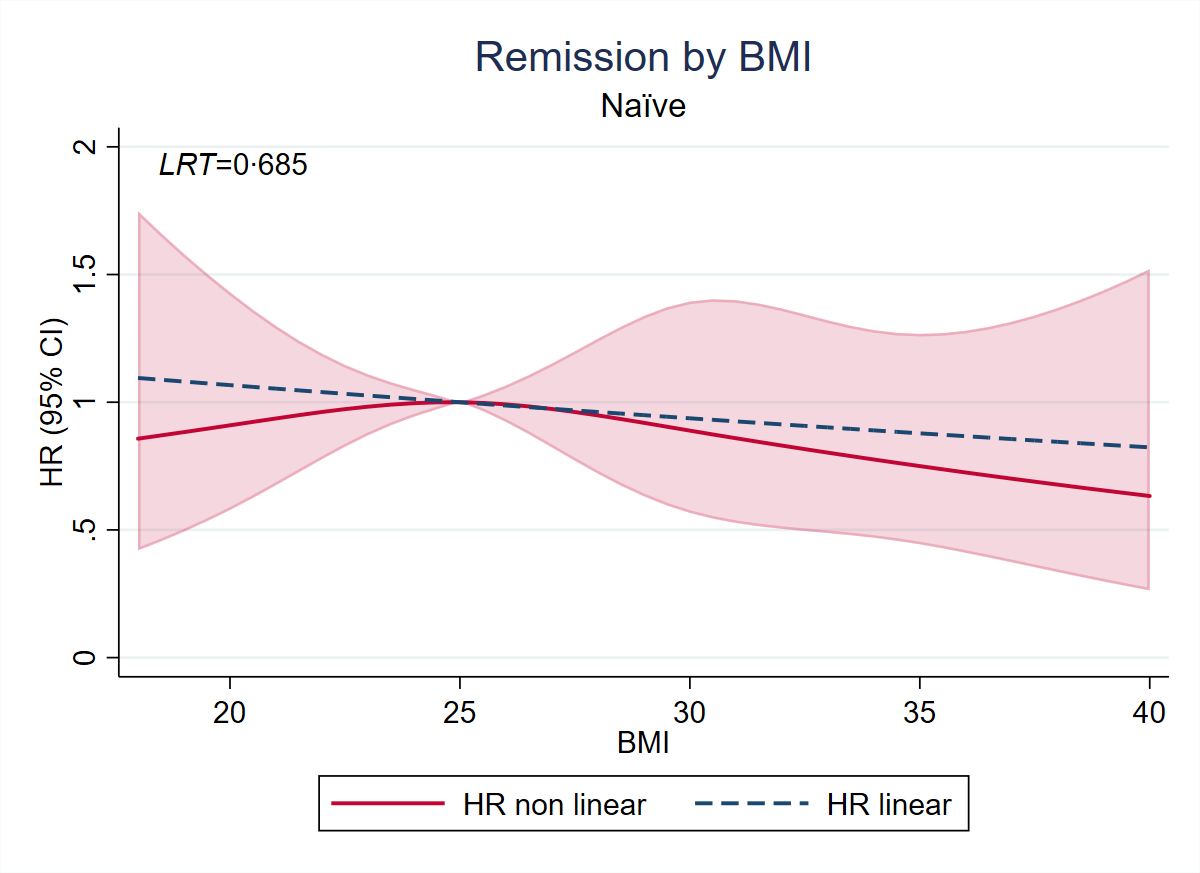

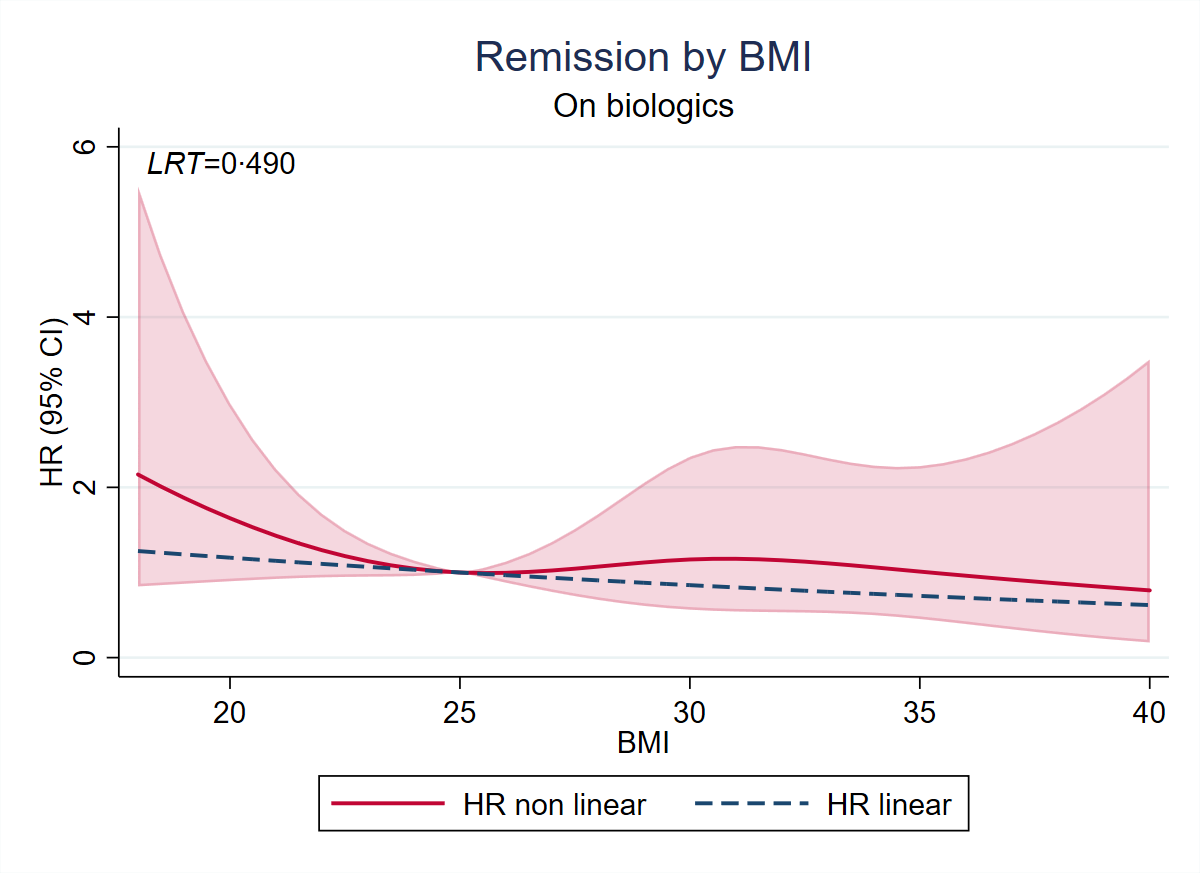
**

BMI = 25 was used as the reference category, as it represented the most frequent value in the study population.

**Online supplement 24. Partial or complete clinical remission stratified by BMI and treatment status.**
Kaplan–Meier curves show the probability of achieving partial or complete clinical remission over time among patients stratified by BMI category (<25; 25-29; ≥30) and biologic treatment status (naïve vs on biologics). The analysis included only patients who had not achieved remission at baseline. Shaded areas indicate 95% confidence intervals.

**
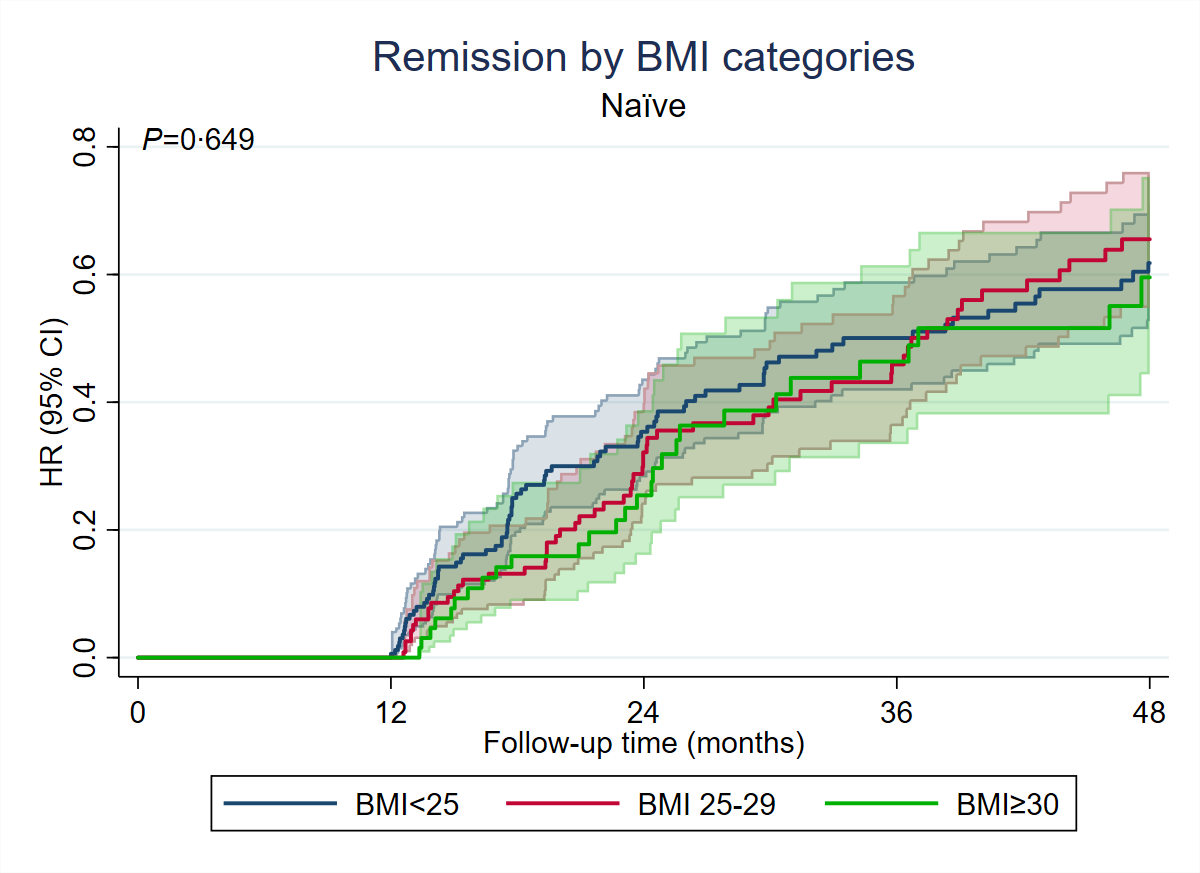

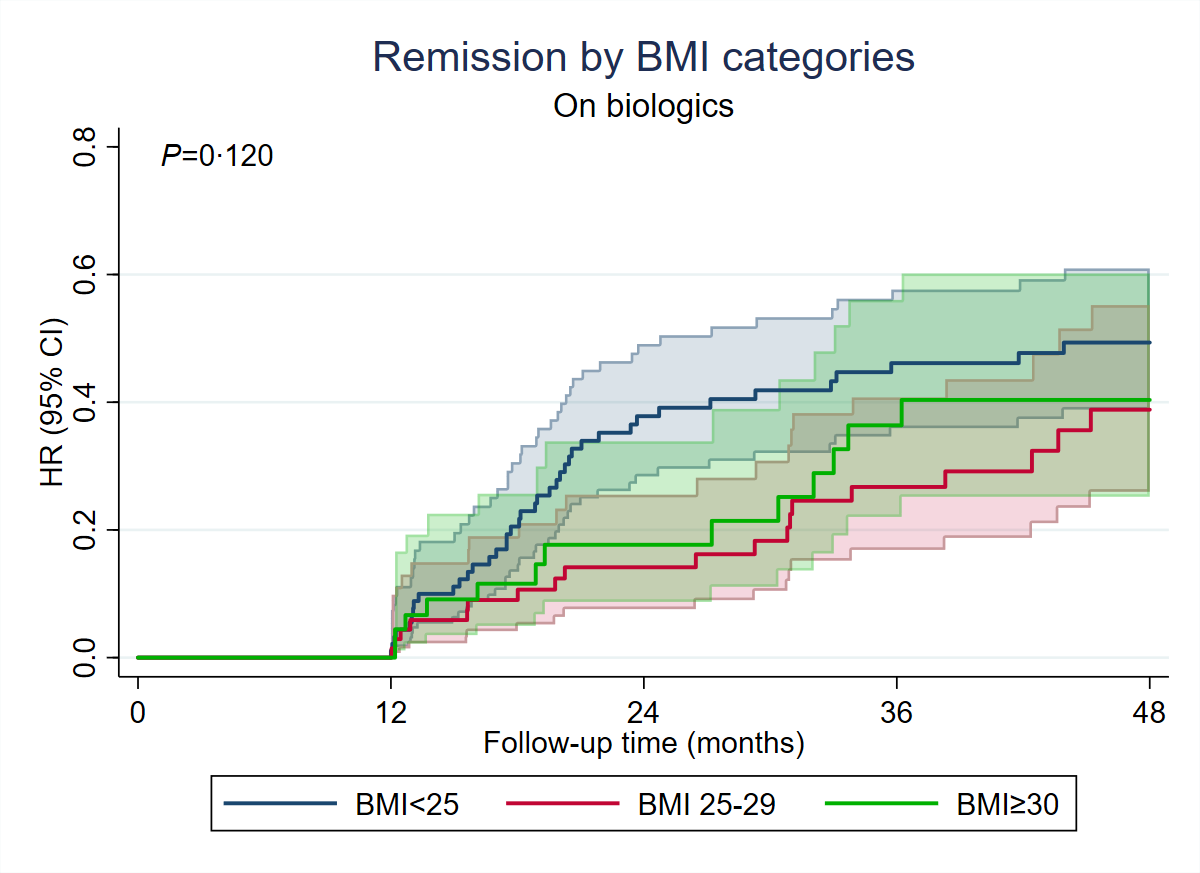
**

**Online supplement 25. Partial or complete clinical remission stratified by BMI and smoking status.**

Kaplan–Meier curves show the probability of achieving partial or complete clinical remission over time among patients stratified by BMI and smoking status (never smokers vs current/former smokers) and biologic treatment status (naïve vs on biologics). The analysis included only patients who had not achieved remission at baseline. Shaded areas indicate 95% confidence intervals.


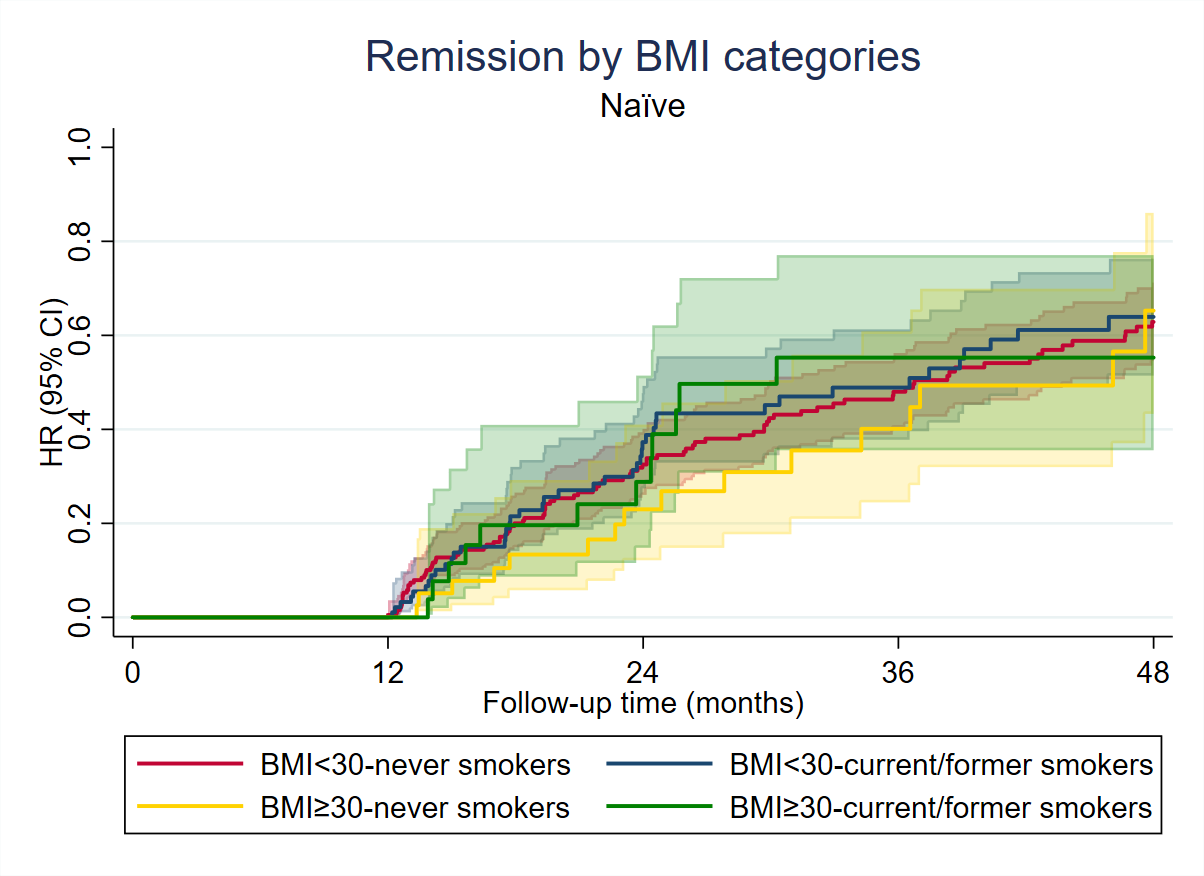

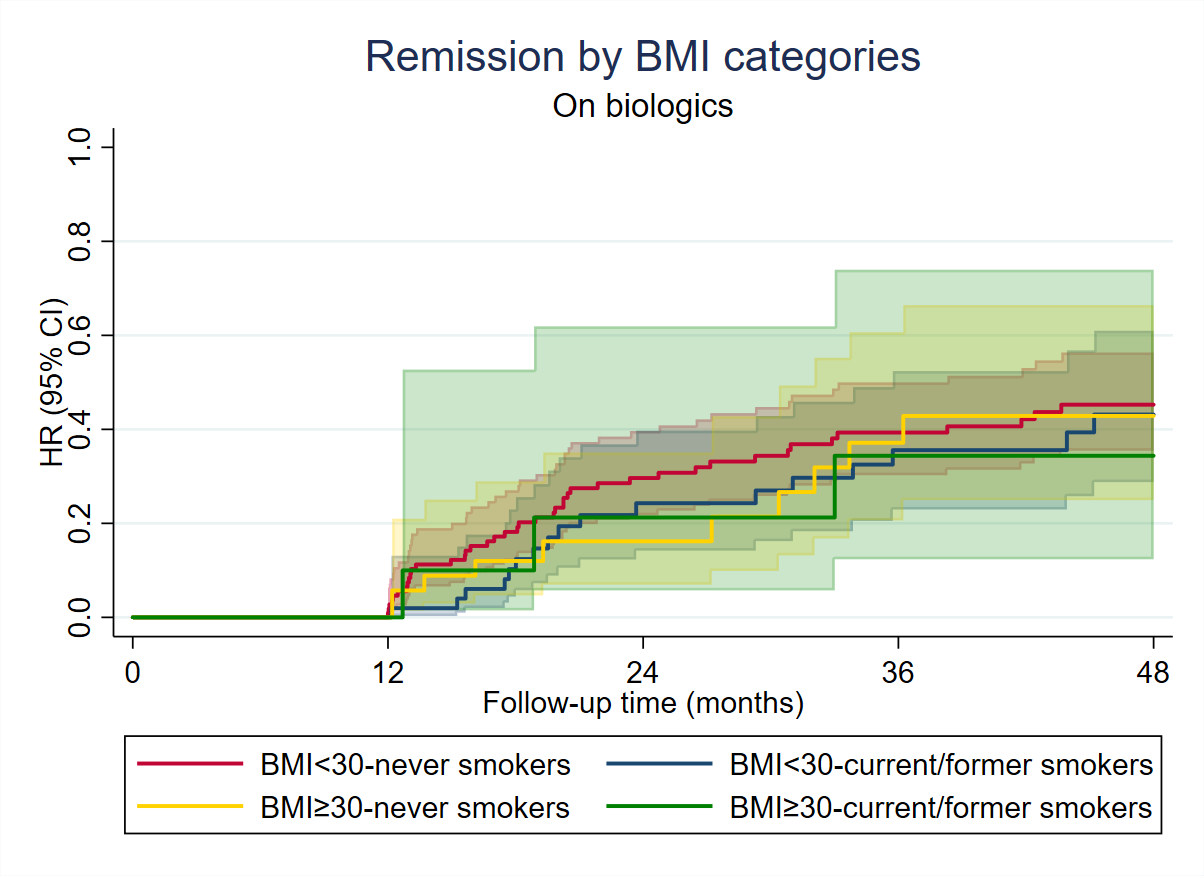


**Online Supplement 26. Multiple imputation–based sensitivity analyses for Cox proportional hazards models.**

*Methods*

To assess the robustness of the primary complete-case Cox analyses and to reduce potential bias related to missingness, multiple imputation by chained equations (MICE) was implemented as a sensitivity analysis. Twenty imputed datasets were generated (m = 20). Continuous variables—including Asthma Control Test (ACT), pre-bronchodilator FEV₁% predicted, and blood eosinophil counts (BEC)—were imputed using predictive mean matching. Exacerbation counts were imputed using Poisson regression, and the dichotomous variable chronic rhinosinusitis with nasal polyps (CRSwNP) was imputed using logistic regression.

### All imputations were conditioned on a set of clinically relevant demographic and disease-related covariates, including age, sex, BMI category, treatment group, visit date, and baseline oral corticosteroid use.

Imputation was applied both to baseline variables and to variables measured at each follow-up time point (ACT, FEV₁, and exacerbation counts). After imputation, partial and complete remission were reconstructed passively within each imputed dataset.

The Cox proportional hazards model specified in the primary analysis was fitted separately within each of the 20 imputed datasets.

*Results*

Across all imputations, effect estimates were highly consistent with those from the complete-case analysis, with minimal between-imputation variability and overlapping confidence intervals, supporting the robustness of the primary findings (appendix p 35).

Depending on the imputed baseline values, a small number of patients could be classified either as already meeting criteria for partial or complete remission and, therefore, excluded from the risk set at time zero, or as not in remission and thus eligible to contribute person-time. This accounts for the slight variation in the numbers reported across the different imputations.

Compared with the complete-case analysis, the naïve multiply imputed analysis included substantially more patients (e.g., 261 additional patients in imputation #1: 743 vs 482), while the multiply imputed on-biologics analysis included 224 additional patients (496 vs 272; in imputation #1) (Appendix p 35).

**Online supplement 27.** **Cox proportional hazards model estimates and numbers at risk across 20 multiply imputed datasets in naïve and on-biologics patients.**

|  |  | **Naive** | | | **On biologics** | | |
| --- | --- | --- | --- | --- | --- | --- | --- |
| **Imputation** |  | **n/N** | **HR (95% CI)** | ***P*** | **n** | **HR (95% CI)** | ***P*** |
| **#1** | **BMI≥30** | 743/920 | 0∙90 (0∙71, 1∙14) | *0∙381* | 496/634 | 0∙85 (0∙64, 1∙14) | *0∙285* |
| **#2** | **BMI≥30** | 747/918 | 0∙91 (0∙71, 1∙16) | *0∙442* | 493/633 | 0∙89 (0∙66, 1∙20) | *0∙434* |
| **#3** | **BMI≥30** | 743/910 | 0∙90 (0∙71, 1∙15) | *0∙413* | 490/622 | 0∙85 (0∙63, 1∙14) | *0∙279* |
| **#4** | **BMI≥30** | 748/922 | 0∙89 (0∙70, 1∙13) | *0∙355* | 508/652 | 0∙88 (0∙66, 1∙19) | *0∙393* |
| **#5** | **BMI≥30** | 756/930 | 0∙90 (0∙70, 1∙14) | *0∙375* | 507/649 | 0∙86 (0∙64, 1∙14) | *0∙292* |
| **#6** | **BMI≥30** | 753/930 | 0∙89 (0∙70, 1∙23) | *0∙331* | 504/648 | 0∙86 (0∙63, 1∙14) | *0∙284* |
| **#7** | **BMI≥30** | 756/931 | 0∙91 (0∙71, 1∙15) | *0∙426* | 504/644 | 0∙86 (0∙64, 1∙16) | *0∙326* |
| **#8** | **BMI≥30** | 751/925 | 0∙89 (0∙70, 1∙13) | *0∙341* | 490/623 | 0∙83 (0∙62, 1∙11) | *0∙217* |
| **#9** | **BMI≥30** | 754/930 | 0∙90 (0∙71, 1∙14) | *0∙392* | 500/637 | 0∙89 (0∙66, 1∙19) | *0∙428* |
| **#10** | **BMI≥30** | 750/924 | 0∙91 (0∙72, 1∙15) | *0∙426* | 508/645 | 0∙86 (0∙64, 1∙15) | *0∙314* |
| **#11** | **BMI≥30** | 754/931 | 0∙91 (0∙71, 1∙14) | *0∙392* | 505/655 | 0∙83 (0∙62, 1∙12) | *0∙230* |
| **#12** | **BMI≥30** | 761/937 | 0∙90 (0∙71, 1∙14) | *0∙380* | 517/673 | 0∙90 (0∙68, 1∙20) | *0∙485* |
| **#13** | **BMI≥30** | 750/922 | 0∙91 (0∙71, 1∙16) | *0∙444* | 492/622 | 0∙85 (0∙64, 1∙14) | *0∙286* |
| **#14** | **BMI≥30** | 750/925 | 0∙91 (0∙72, 1∙16) | *0∙451* | 515/667 | 0∙89 (0∙73 1∙20) | *0∙450* |
| **#15** | **BMI≥30** | 744/916 | 0∙90 (0∙71, 1∙14) | *0∙389* | 489/619 | 0∙88 (0∙66, 1∙18) | *0∙401* |
| **#16** | **BMI≥30** | 749/918 | 0∙88 (0∙70, 1∙12) | *0∙321* | 498/634 | 0∙91 (0∙68, 1∙22) | *0∙542* |
| **#17** | **BMI≥30** | 754/931 | 0∙89 (0∙70, 1∙13) | *0∙340* | 504/639 | 0∙86 (0∙65, 1∙16) | *0∙329* |
| **#18** | **BMI≥30** | 754/930 | 0∙89 (0∙70, 1∙13) | *0∙324* | 508/647 | 0∙89 (0∙67, 1∙19) | *0∙430* |
| **#19** | **BMI≥30** | 755/930 | 0∙90 (0∙71, 1∙14) | *0∙391* | 508/643 | 0∙86 (0∙64, 1∙15) | *0∙320* |
| **#20** | **BMI≥30** | 750/919 | 0∙89 (0∙70, 1∙13) | *0∙348* | 496/627 | 0∙91 (0∙68, 1∙21) | *0∙517* |

Patients not in remission (at risk): n (with follow-up) / N (with or without follow-up).

Variables with missing observations imputed at baseline included CRSwNP (Naïve: 2 [0.18%], On biologics: 10 [1.02%]), blood eosinophil count (Naïve: 168 [15%], On biologics: 250 [25.5%]), FEV₁% predicted (Naïve: 299 [26.7%], On biologics: 273 [27.8%]), ACT score (Naïve: 129 [11.5%], On biologics: 111 [11.3%]), and exacerbation (Naïve: 76 [6.8%], On biologics: 67 [6.8%]).
