# Supplementary material for: Clinical outcomes and remission trajectories in obese and non-obese patients with severe asthma treated with biologics: a retrospective longitudinal cohort study from the Severe Asthma Network Italy (SANI) registry
Source: Lancet Reg Health Eur. 2026 May 7;66:101695. doi: 10.1016/j.lanepe.2026.101695 (PMC13186060; doi:10.1016/j.lanepe.2026.101695)
Supplement: SANI network [file mmc2.pdf]

Centro SANI	PI surname	PI name
Clinica malattie respiratorie e allergologia IRCCS- AOU SanMartino - Uni - Ge	Bagnasco	Diego
AO Allerg. Immuno. Mauriziano- Torino	Brussino	Luisa
UO di Clinica Pneumologica SUN - Presidio Ospedaliero Ospedaledei Colli - I	Calabrese	Cecilia
Ambulatorio dellasma. Diagnosi e trattamento dellasma grave- A. O.U. Careg	Camiciottoli	Gianna
USD Allergologia - Ospedale Borgo Roma - Verona	Caminati	Marco
Malattie dellapparato respiratorio, policlinico di Bari	Carpagnano	Giovanna Elisiana
UOC Allergologia - Fondazione Policlinico Gemelli- Roma	Caruso	Cristiano
IRCCS Policlinico S. Matteo - Pavia	Corsico	Angelo Guido
Allergologia, Immunologia Clinica e Reumatologia - ASST Mantova	Costantino	Maria Teresa
Pneumologia Riabilitativa e Allergologia - AOU Policlinico VittorioEmanuele -	Crimi	Claudia
IRCCS Ca Granda - Fondazione Ospedale Maggiore Policlinico -Milano	D'Adda	Alice
UOC ALLERGOLOGIA - PO CIVITANOVA MARCHE - AREA VASTA3	D'Alò	Simona
Respiratory Department- AO Dei Colli Napoli	D'Amato	Maria
Ambulatorio Asma Grave presso U.O.C di Pneumologia -ARNAS Garibaldi, CT	D'Amico	Leda
Struttura Dipartimentale di Allergologia ed Immunologia ClinicaPOC SS Annu	D'Andria	Corrado
Allergologia e Immunologia Clinica - Policlinico Universitario di Cagliari	Del Giacco	Stefano
Ospedale Papa Giovanni - Bergamo	Di Marco	Fabiano
Centro Asma e Allergologia- SC Pneumologia - Arcispedale S. MariaNuova IR	Facciolongo	Nicola Cosimo
SOS Allergologia e Immunologia Prato	Farsi	Alessandro
U.O.C. Pneumologia Ospedale delle Apuane - Massa	Latorre	Manuela
S.C. Pneumologia dellOspedale S. Corona Pietra Ligure - P.O. delPonente	Milanese	Manlio
U.O. Pneumologia, ASST Santi Paolo e Carlo - Milano	Mondoni	Michele
U.O. Allergologia ed Immunologia Clinica - AO UniversitariaPoliclinico di Bari	Nettis	Eustachio
Centro Aziendale per la Cura delle Malattie Allergiche eImmunologiche Grav	Patella	Vincenzo
Dipartimento di Scienze Mediche e Chirurgiche Università MagnaGraecia - (	Pelaia	Girolamo
Ambulatorio Asma Grave - UO Medicina Apparato Respiratorio -Spedali civili	Pini	Laura
UOC ALLERGOLOGIA E IMMUNOLOGIA CLINICA - AOUPOLICLINICO G. MARTIN(	Ricciardi	Luisa
SSDU Asma Grave e Malattie Rare del Polmone San Luigi Gonzaga- Orbassa	Ricciardolo	Fabio Luigi Massimo
S. C. Pneumologia - Università Cattolica - P. Gemelli - Roma	Richeldi	Luca
Allergologia e Immunologia Clinica, Dipartimento di ScienzeCliniche e Sperir	Ridolo	Erminia
Reparto di Pneumologia Ospedale Sacco - Milano	Santus	Pierachille
AOU Policlinico Paolo Giaccone - Palermo	Scichilone	Nicola
S. C. Malattie dellApparato Respiratorio 4a Direz. Univ. A. O. U.Ospedali Riun	Scioscia	Giulia
UOC Pneumologia Riabilitativa - Fondazione S. Maugeri IstitutoScientifico -	Spanevello	Antonio
SC Broncopneumotisiologica ASST Grande OspedaleMetropolitano Niguarda	Tarsia	Paolo
U.O. Diagnosi e Terapia delle malattie allergiche e dell'immunitario -Salerno	Triggiani	Massimo
Divisione di Allergologia ed Immunologia Clinica, AOU Federico IINapoli	Varricchi	Gilda
Unit of Immunology, Rheumatology, Allergy and Rare DiseasesIRCCS San Raf	Yacoub	Mona-Rita
